# Understanding the Epidemiology of Multi-Drug Resistant Gram-Negative Bacilli in the Middle East Using a One Health Approach

**DOI:** 10.3389/fmicb.2019.01941

**Published:** 2019-08-23

**Authors:** Iman Dandachi, Amer Chaddad, Jason Hanna, Jessika Matta, Ziad Daoud

**Affiliations:** ^1^Faculty of Medicine and Medical Sciences, Clinical Microbiology Laboratory, University of Balamand, Beirut, Lebanon; ^2^Division of Clinical Microbiology, Saint George Hospital University Medical Center, Beirut, Lebanon

**Keywords:** colistin, ESBL, carbapenemases, one health, MDROs

## Abstract

In the last decade, extended-spectrum cephalosporin and carbapenem resistant Gram-negative bacilli (GNB) have been extensively reported in the literature as being disseminated in humans but also in animals and the environment. These resistant organisms often cause treatment challenges due to their wide spectrum of antibiotic resistance. With the emergence of colistin resistance in animals and its subsequent detection in humans, the situation has worsened. Several studies reported the transmission of resistant organisms from animals to humans. Studies from the middle east highlight the spread of resistant organisms in hospitals and to a lesser extent in livestock and the environment. In view of the recent socio-economical conflicts that these countries are facing in addition to the constant population mobilization; we attempt in this review to highlight the gaps of the prevalence of resistance, antibiotic consumption reports, infection control measures and other risk factors contributing in particular to the spread of resistance in these countries. In hospitals, carbapenemases producers appear to be dominant. In contrast, extended spectrum beta lactamases (ESBL) and colistin resistance are becoming a serious problem in animals. This is mainly due to the continuous use of colistin in veterinary medicine even though it is now abandoned in the human sphere. In the environment, despite the small number of reports, ESBL and carbapenemases producers were both detected. This highlights the importance of the latter as a bridge between humans and animals in the transmission chain. In this review, we note that in the majority of the Middle Eastern area, little is known about the level of antibiotic consumption especially in the community and animal farms. Furthermore, some countries are currently facing issues with immigrants, poverty and poor living conditions which has been imposed by the civil war crisis. This all greatly facilitates the dissemination of resistance in all environments. In the one health concept, this work re-emphasizes the need to have global intervention measures to avoid dissemination of antibiotic resistance in humans, animals and the environment in Middle Eastern countries.

## Introduction

In the 1940s, the discovery of antibiotics was seen as one of medicine’s major achievements that saved millions of lives (van Hoek et al., 2011). However, in the last decade antimicrobial resistance has significantly increased in bacteria and reduced the effectiveness of many clinically important antibiotics (Seiffert et al., 2013). Gram-negative bacilli (GNB) are among the most common causative agents of infectious diseases (Tian et al., 2012). Members of this family are ubiquitous, i.e., can be found in humans and animals’ intestinal microflora, but also in the environment (Verraes et al., 2013). Among other resistant organisms, GNB are distinct in view of their complex mechanisms of resistance. These are mainly mediated via the production of extended spectrum beta lactamases (ESBL), AmpC and carbapenemases (Schill et al., 2017). These hydrolyzing enzymes confer bacterium resistance toward the most common class of antibiotics prescribed nowadays in clinical settings: beta lactams (Ruppe et al., 2015). Furthermore, resistance genes of these enzymes are often located on plasmids harboring resistance determinants to other classes of antibiotics; thus challenging therapeutic options when infectious diseases do occur (Ruppe et al., 2015). The dissemination GNB resistant to extended spectrum cephalosporins and carbapenem, necessitates the re-use of colistin (a polymyxin E antibiotic) previously abandoned due to its toxicity and side effects (Olaitan et al., 2014b). The re-introduction of colistin in recent years has also seen the emergence of resistance, further complicating the clinical situation. Colistin resistance occurs either via chromosomal mutations that mediates the alteration of the lipid A moiety of the lipopolysaccharide chain (Baron et al., 2016); or via the acquisition of an *mcr* gene (Olaitan et al., 2016a).

Previously known to be confined to the hospital settings, multi-drug resistant organisms (MDROs) are nowadays widely spread in animals and the environment (Rafei et al., 2015a). Dandachi et al. (2018a) reported the wide dissemination of ESBL producers as well as colistin resistant GNB in poultry, cattle, swine and companion animals in Mediterranean countries. For instance, several studies have shown that multi-drug resistance (MDR) can be readily transferred from one ecosystem to another via direct/indirect contact with contaminated animals and/or animal products (Huijbers et al., 2014), dust (Blaak et al., 2015), air (von Salviati et al., 2015), contaminated wastewaters (Guenther et al., 2011), and soil fertilized with animal manure (Laube et al., 2014). Humans, animals, and the environment together therefore form an interconnected system that should be carefully addressed in terms of bacterial resistance, antibiotic stewardship, and infection control measures.

In this context, Middle Eastern countries are thus of special interest. The dissemination of MDROs in this region of the world involves an interplay of over/misuse of antibiotics in humans and animals, the absence of infection control measures and most importantly the recent continuous population mobilization due to socio-economic conflicts and multiple war crises. In this review, our aim is to describe the epidemiology of extended spectrum cephalosporin, carbapenem and colistin resistant GNB in humans, animals and the environment in the Middle Eastern area. The Middle East includes 15 countries: Bahrain, Egypt, Iraq, Iran, Jordan, the Kingdom of Saudi Arabia (KSA), Kuwait, Lebanon, Palestine, Qatar, Sultanate Oman, Syria, Turkey, the United Arab Emirates (UAE), and Yemen. Our attempt is to highlight the gaps in bacterial resistance reports, antibiotic consumption data as well as infection control measures in this distinct area of the world.

## Distribution of Multi-Drug Resistant Organisms in Humans

### Infections With ESBL/AmpC Producers

**FIGURE 1:**
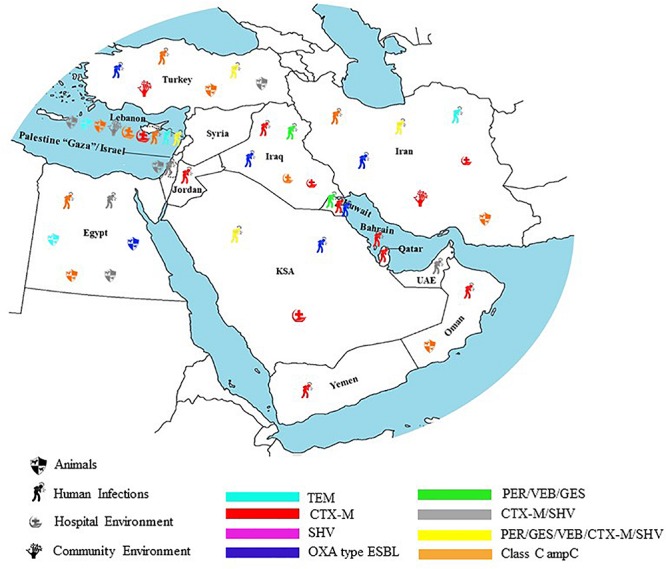
Geographical distribution of ESBL in humans, animals, hospital, and community environment in the Middle East.

Extended spectrum cephalosporins and penicillin’s have been widely used in clinical settings due to their wide spectrum of activity as well as their negligible toxicity compared to other antimicrobial agents (Bush and Bradford, 2016). Their un-restricted use by physicians, in addition to their purchasing ease, without medical prescription in the community pharmacies, plays an important role in the emergence of bacteria resistant to these antimicrobial agents (Figure 1).

In Iran, studies have shown that in *Klebsiella* spp., resistance to extended spectrum beta lactams is mainly mediated via the production of CTX-M variants (CTX-M-15, CTX-M-3, CTX-M-8, CTX-M-1, and CTX-M-2) (Feizabadi et al., 2010; Peerayeh et al., 2014; Bialvaei et al., 2016; Akya et al., 2018; Dehshiri et al., 2018) and to a lesser degree via SHV (SHV-12, SHV-11, SHV-5) (Feizabadi et al., 2010; Shakib et al., 2018), TEM genes (Gholipour et al., 2014; Maleki et al., 2018) and others (VEB, PER, and GES) (Sedighi et al., 2017). pAmpC beta lactamase genes were reported by two studies in clinical isolates of *Klebsiella pneumoniae* (Mansouri et al., 2012; Kiaei et al., 2018). PFGE and ERIC-PCR analysis in these studies showed the presence of different clones in each clinical center (Akya et al., 2018; Hashemizadeh et al., 2018; Kiaei et al., 2018; Maleki et al., 2018). This is with the exception of one study where high clonal relatedness among ESBL *K. pneumoniae* strains was reported (Ghaffarian et al., 2018). Mehrgan et al. (2010) showed that intensive care unit (ICU) or medical ward stays are significantly associated with the acquisition of ESBL *Klebsiella* spp. Indeed, these resistant organisms are often found in patients who are very young and who have not yet developed full immunity, thus making them susceptible to opportunistic pathogen infections (Mehrgan et al., 2010). Similarly, to *Klebsiella* spp., CTX-M-15 followed by SHV, TEM and to a lesser extent CIT, were the most common beta lactamase genes detected in clinical strains of *Escherichia coli* (Gholipour et al., 2014; Rezai et al., 2015; Shayan and Bokaeian, 2015; Bialvaei et al., 2016). It is worth noting the detection of CTX-M, TEM, and CIT beta lactamases in diarrheagenic *E. coli* strains: Enteroaggregative and Enteropathogenic ones (Heidary et al., 2014; Aminshahidi et al., 2017). These *E. coli* patotypes are always pathogenic when present in human intestines (Fratamico et al., 2016). Moreover, one recent study reported the isolation of CTX-M-15 extraintestinal pathogenic *E. coli* ST131 from inpatients and outpatients in Iran (Namaei et al., 2017). Statistical analysis indicated that ESBL producing ST131 *E. coli* strains were positively associated with CTX-M variants, CTX-M-15, and TEM beta lactamases (Namaei et al., 2017). Moreover, strictly pathogenic species producing ESBL were also detected: CTX-M-1/CTX-M-15 *Salmonella* spp. and CTX-M-15/CMY-2 *Shigella* spp. (Salimian Rizi et al., 2015; Bialvaei et al., 2016; Aminshahidi et al., 2017). The high incidence of cephalosporins resistance in pathogenic bacteria in this country may be attributed in part to their inappropriate and high use in clinical settings (Amin et al., 2018); this is in addition to their extensive utilization in the Iranian community via self-medication (SM) (Zamanlou et al., 2018). Other ESBL producers that have been detected in clinical settings in Iran include CTX-M-15/TEM-169, SHV-12 producing *Enterobacter cloacae* (Peymani et al., 2014). On the other hand, in *Pseudomonas aeruginosa*, the major ESBL types were OXA-10/OXA-4, PER-1, VEB-1, and GES-1 (Mirsalehian et al., 2010; Alikhani et al., 2014; Farshadzadeh et al., 2014; Emami et al., 2015; Davodian et al., 2016; Amirkamali et al., 2017). This is followed by CTX-M, TEM-116, SHV-12, DHA and hyper-produced ADC enzymes (Bokaeian et al., 2014; Fazeli and Momtaz, 2014; Rafiee et al., 2014). Only one study in Iran revealed 13 distinct profiles among 100 ESBL/carbapeneme resistant *P. aeruginosa* isolated from burn patients via RAPD analysis. A dominant RAPD type was observed consisting of 80 isolates, thus revealing the possible existence of endemic clones circulating among patients (Neyestanaki et al., 2014).

In Turkey, ESBL production in *E. coli* and *K. pneumoniae* is mainly mediated via CTX-M-group1 (CTX-M-15 and CTX-M-1) and CTX-M-group2. Others include PER and OXA-10 in *E. coli* (Elaldi et al., 2013; Gorgec et al., 2015; Iraz et al., 2015). Furthermore, CMY-2, CIT, MOX, EBC, FOX, and ACT-1 have been detected in *E. coli* and *Klebsiella* spp., respectively (Demirbakan et al., 2008; Sari et al., 2013; Yilmaz et al., 2013). PFGE analysis showed no major clonal relationship per species in each clinical center (Durmaz et al., 2015; Gorgec et al., 2015). Multivariate analysis showed that urinary catheter insertion was a common risk factor for acquiring an infection with an ESBL quinolone resistant *E. coli* strain in inpatients and outpatients alike (Durmaz et al., 2015). Moreover, in two other studies the risk factors for the development of an ESBL *K. pneumoniae* blood stream infections were high, with the duration of hospitalization being a common factor (Serefhanoglu et al., 2009). Other factors included prior antibiotic use and the use of aminoglycosides (Tanir Basaranoglu et al., 2017). Other ESBL producing Enterobacteriaceae detected in Turkey include CTX-M/TEM/SHV/qnrA aac(6′)-ib *Enterobacter* spp. and VEB-1/qnrA1 *Providencia stuartii* (Nazik et al., 2009, 2011; Erdem et al., 2018). Interestingly, Agin et al. (2011) reported an outbreak of *Salmonella enterica* serovar typhimurium producing SHV-12 and CTX-M-3 ESBLs. In view of this, handwashing and disinfection procedures in addition to the establishment of an active surveillance program were initiated. These infection control measures led to the containment of the outbreak after 2 years. As for non-fermenters, PER-1 was the main ESBL type detected in *P. aeruginosa* and *Acinetobacter* spp. alike (Atilla et al., 2012; Erac et al., 2013; Keskin et al., 2014). In *Pseudomonas* spp. additional types were also detected such as OXA-10, OXA-14, and GES-1 (Aktas et al., 2012; Er et al., 2015).

In Lebanon, clinical epidemiological studies showed the predominance of CTX-M-15 and SHV-5a in *E. coli* and *K. pneumoniae* (Charrouf et al., 2014; Daoud et al., 2017). Furthermore, one report described the presence of SHV-11/CTX-M-15/acc(6′)-lb-cr/qnrb1 producing ST336 *K. pneumoniae* (Tokajian et al., 2015). PFGE analysis revealed clonal diversity among ESBL producing *E. coli* and *K. pneumoniae* (Daoud et al., 2017). As for the effect of antibiotic prescription and its correlation with the level of bacterial resistance, Daoud et al. (2017) found a significant association between aztreonam resistance and the use of penicillin’s, and between cefuroxime, ceftazidime, cefoxitin, ciprofloxacin resistance and 3rd/4th generation cephalosporins use in *Klebsiella* spp. Moreover, one study reported the detection of four unrelated ESBL producing *Shigella sonnei* isolated from the stool samples of patients admitted for bacillary dysentery. These isolates harbored the CTX-M-15 gene on the plasmid and were flanked by ISEcp1 (Sabra et al., 2009).

In Israel, one study found low prevalence of ESBL producers in a clinical center (Chazan et al., 2009). The authors suggest that one of the reasons for this finding is the strict supervision of antibiotic prescription applied in their hospital; in addition to the limited use of ceftazidime (Chazan et al., 2009). Another study in the same country, argued that recent hospitalization, urinary tract infection (UTI) prophylaxis and *Klebsiella* spp. UTI are risk factors for the development of community acquired ESBL UTI (Dayan et al., 2013). Another has found that prior antipseudomonal therapy and empirical cephalosporin therapy are independent risk factors for UTI, caused by an ESBL producing *Proteus mirabilis* (Cohen-Nahum et al., 2010). As for the underlying genes of resistance, one study showed the presence of CTX-M-2, CTX-M-15, and CTX-M-14 in predominantly ST131 *E. coli* strains (Karfunkel et al., 2013). In this study, 93 and 51% of the isolates were co-resistant to fluoroquinolones and gentamicin, respectively. Transformation experiments suggest that aminoglycosides resistance is co-carried on the same plasmid harboring the CTX-M gene (Karfunkel et al., 2013). Other studies in Palestine have found clonal diversity among ESBL producing *E. coli* clinical strains (Adwan et al., 2014; Tayh et al., 2016). On the other hand, in Israel, Karfunkel et al. (2013) reported the dominance of the ST131 among 41 CTX-M positive *E. coli* strains isolated for community onset bacteremia (COBSI) at Tel Aviv Sourasky Medical Center. In this center, the incidence of COBSI has increased 2.7-fold over a 7 year period. This increase appears to be correlated with the clonal expansion of ST131 *E. coli* strains carrying the blaCTX-M-14 and blaCX-M-15 genes (Karfunkel et al., 2013). ESBL production by *K. pneumoniae* in clinical settings was reported, whereby CTX-M-15, CTX-M-14a, CTX-M-3, SHV-12, SHV-5, and SHV-33 were detected (Tayh et al., 2017). In Jordan, very few studies have addressed the prevalence of ESBL producers in clinical settings. However, blaCTX-M (CTX-M-15, CTX-M-1, and CTX-M-9) was the only ESBL type detected in Enterobacteriaceae notably *E. coli* and ST131 *K. pneumoniae* strains (Hayajneh et al., 2015; Aqel et al., 2017; Nairoukh et al., 2018).

In Iraq, CTX-M-1, SHV, TEM producing *E. coli* strains were reported in recurrent UTI patients. In this report, MDR was significantly higher in ESBL *E. coli* versus non-ESBL ones (Al-Mayahie and Al Kuriashy, 2016). Similar results were obtained in a study addressing ESBL producers in pregnant/non-pregnant women with symptomatic genital tract infection. It is worth mentioning that ESBL producers co-resistant to non-beta lactam antibiotics is of special interest in this category; this is in view of the narrow choice of antibiotics that could be used in this category of patients (Al-Mayahie, 2013). Furthermore, in this country, CTX-M, SHV, TEM, and OXA ESBLs were described in clinical isolates of *Morganella morganii* with high resistance toward minocycline, trimethoprim- sulfamethoxazole and ciprofloxacin (Al-Muhanna et al., 2016). In parallel, VEB, PER, and OXA-10 were detected in high risk strains of *P. aeruginosa*: ST244, ST235, ST308, and ST654 (van Burgh et al., 2018).

In Kuwait, diverse genetic profiles of ESBL producing *E. coli* strains were detected in inpatients and outpatients alike (Dashti et al., 2014). CTX-M-15 followed by SHV-12, CMY-2, CTX-M-14, CTX-M-56, and CTX-M-2 are the most common ESBL types detected (Dashti et al., 2014; Jamal et al., 2015). In contrast to *E. coli*, one study in Kuwait reported identical PFGE profiles of *K. pneumoniae* SHV-112 positive strains isolated from blood and urine specimens of ICU patients (Dashti et al., 2010a). Another study however, reported different sequence types of *K. pneumoniae* detected in hospitalized patients: ST677, ST16, ST107, and ST485 producing CTX-M-15, SHV-11, and CTX-M-14 beta lactamases (Jamal et al., 2015).

In KSA, ST131 followed by ST38 *E. coli* strains producing ESBL appears to be predominant in clinical settings (Alghoribi et al., 2015; Alyamani et al., 2017; Yasir et al., 2018). In these, the main ESBL types detected were: CTX-M-15, CTX-M-9, CTX-M-1, CTX-M-8/25, CTX-M-2, CTX-M-14, SHV-12, and SHV-5 (Shibl et al., 2012; Al Sheikh et al., 2014; Alyamani et al., 2017; Yasir et al., 2018). Indeed, one study has shown that ESBL producers were significantly more resistant to aminoglycosides, ciprofloxacin and trimethoprim-sulfamethoxazole (Hassan and Abdalhamid, 2014). Al-Otaibi and Bukhari (2013) found that recurrent UTIs, surgical intervention, children with vesicoureteric reflux and patients who had underlying renal transplant and renal disease are all possible risk factors for the acquisition of an ESBL UTI *E. coli* strain.

As for ESBL *K. pneumoniae*, the situation appears to be similar to their *E. coli* counterparts (Ahmad et al., 2009; Hassan and Abdalhamid, 2014; Somily et al., 2014). This is with the exception to the additional detection of other CTX-M variants such as CTX-M-3, CTX-M-82, CTX-M-57, and CTX-M-27 in *K. pneumoniae* as compared to *E. coli* strains (Al-Qahtani et al., 2014). In addition, in view of the wide diversity of ESBL *K. pneumoniae* isolates, it seems that clonal spread plays a negligible role in the dissemination of these strains (Al-Qahtani et al., 2014). Moreover, one study reported the detection of CTX-M-14 and SHV-12 in clinical isolates of *Citrobacter freundii* and *Enterobacter* spp. (Al Sheikh et al., 2014). SHV-5, CMY-2, and DHA-1 were also detected in *Enterobacter* spp. isolated from clinical settings in KSA (Abdalhamid et al., 2017a). On the other hand, VEB, GES, and OXA-10 were detected in *P. aeruginosa* clinical strains (Al-Agamy et al., 2012; Tawfik et al., 2012).

ESBL production in *Acinetobacter baumannii* on the other hand, was meditated via CTX-M and GES variants (Alyamani et al., 2015; Al-Agamy et al., 2017). Similarly, to other ESBL producing GNB in KSA, MLST analysis revealed the presence of a wide variety of sequence types in ESBL *A. baumannii* strains (Alyamani et al., 2015; Al-Agamy et al., 2017) (Table 1). Moreover, one study addressing the hajj pilgrims of Marseille, reported the detection of 2 CTX-M-2 producing *Salmonella* spp. Both strains were gentamicin and colistin resistant, in addition, they belonged to the epidemic Newport serotype ST45 (Olaitan et al., 2015). This finding calls for improved public health surveillance during the Hajj period in order to prevent the dissemination of MDROs in KSA and worldwide (Olaitan et al., 2015).

**TABLE 1:** Sequence and plasmid types associated with ESBL genes in humans, animals, and environment in the Middle East.

**Country**	**ESBL gene**	**Reservoir**	**Species**	**Sequence type/phylogroup**	**Plasmid type**
Iran	CTX-M	Humans	*E. coli*		
			*K. pneumoniae*		
			*E. cloacae*		
			*Salmonella* spp.		
			*Shigella* spp.		
		Hospital environment	*E. coli*		
		Community environment	*A. baumannii*		
	TEM	Humans	*E. coli*		
			*K. pneumoniae*		
			*E. cloacae*		
			*A. baumannii*		
			*P. aeruginosa*		
		Hospital environment	*E. coli*		
			*P. aeruginosa*		
		Community environment	*A. baumannii*		
	SHV	Humans	*E. coli*		
			*K. pneumoniae*		
			*E. cloacae*		
			*P. aeruginosa*		
		Animals	*E. coli*		
		Hospital environment	*E. coli*		
			*P. aeruginosa*		
	OXA	Humans	*E. coli*		
			*P. aeruginosa*		
		Hospital environment	*E. coli*		
	GES	Humans	*K. pneumoniae*		
			*P. aeruginosa*		
	VEB	Humans	*E. coli*		
			*K. pneumoniae*		
			*P. aeruginosa*		
	PER	Humans	*K. pneumoniae*		
			*A. baumannii*		
			*P. aeruginosa*		
Turkey	CTX-M	Humans/community environment	*E. coli*	–	
		Animals	*E. coli*	A, D, B1, B2	
		Humans	*K. pneumoniae*		
		Humans	*Enterobacter* spp.		
	TEM	Humans/animals	*E. coli*		
		Humans	*K. pneumoniae*		
		Humans	*Enterobacter* spp.		
	SHV	Humans	*E. coli*		
			*Enterobacter* spp.		
			*S. paratyphi*		
		Animals	*E. coli*	A, D, B1, B2	
		Community environment	*E. coli*		
	OXA	Humans	*E. coli*		
			*P. aeruginosa*		
		Community environment	*E. coli*		
	GES	Humans	*P. aeruginosa*		
	VEB	Humans	*K. pneumoniae*		
	PER	Humans	*E. coli*		
			*P. aeruginosa*		
			*A. baumannii*		
Lebanon	CTX-M	Humans	*E. coli*		
			*K. pneumoniae*		
			*Shigella* spp.		
		Animals	*E. coli*	ST10, ST617, ST58, ST69, ST1303, ST156, ST5470, ST354, ST155, ST3224	
		Community environment	*E. coli*	ST328, ST405, ST34, ST48, ST131, ST120, ST2067, ST10, ST38, ST410	IncFII
				ST38, ST1431, ST46	IncFIA
				ST212	IncY
				ST617, ST4144, ST6470, ST6222, ST90, ST38, ST4608, ST6894, ST127	
			*K. pneumoniae*	ST22, ST336, ST15, ST16	IncFIIk
			*C. freundii*		IncY
			*C. braakii*		IncY
		Hospital environment	*E. coli*		
			*K. pneumoniae*		
	TEM	Humans	*E. coli*		
			*K. pneumoniae*		
		Animals	*E. coli*		
			*K. pneumoniae*		
		Hospital environment	*E. coli*		
			*K. pneumoniae*		
	SHV	Humans	*E. coli*		
			*K. pneumoniae*		
		Animals	*E. coli*		
			*K. pneumoniae*		
		Community environment	*E. coli*	ST46	IncFII
				ST617, ST6470, ST90, ST4608, ST6480, ST4144	
	SHV	Hospital environment	*E. coli*		
			*K. pneumoniae*		
	VEB	Humans	*K. pneumoniae*		
Palestine/Israel	CTX-M	Humans	*E. coli*	ST1, ST2, ST39, ST131, ST472, ST473, ST474, ST475, ST476, ST477	
			*K. pneumoniae*		
		Animals	*E. coli*		
	TEM	Humans	*E. coli*		
	SHV	Humans	*K. pneumoniae*		
		Animals	*E. coli*		
Jordan	CTX-M	Humans	*E. coli*	ST131	
			*K. pneumoniae*	A2, C	
Iraq	CTX-M	Humans	*E. coli*		
			*M. morganii*		
	TEM	Humans	*M. morganii*		
	SHV	Humans	*E. coli*		
			*M. morganii*		
	OXA	Humans	*P. aeruginosa*	ST244, ST308	
	VEB	Humans	*P. aeruginosa*	ST235	
	PER	Humans	*P. aeruginosa*		
Bahrain	CTX-M	Humans	*E. coli*		
			*K. pneumoniae*		
	TEM	Humans	*E. coli*		
			*K. pneumoniae*		
	SHV	Humans	*E. coli*		
Qatar	CTX-M	Humans	*E. coli*		
			*K. pneumoniae*		
	TEM	Humans	*E. coli*		
			*K. pneumoniae*		
	SHV	Humans	*E. coli*		
United Arab Emirates	CTX-M	Humans	*E. coli*	ST131	IncFII-FIA-FIB
				ST131	
	SHV	Humans	*K. pneumoniae*		
Oman	CTX-M	Humans	*E. coli*		
Yemen	CTX-M	Humans	*E. coli*	ST131	
			*K. pneumoniae*	ST1399, ST340, ST405, ST147, ST29	
	SHV	Humans	*K. pneumoniae*	ST1399, ST340, ST405, ST147	
Kuwait	CTX-M	Humans	*E. coli*	ST131, ST405, ST38	
			*K. pneumoniae*		
	SHV	Humans	*E. coli*	ST131	
			*K. pneumoniae*		
KSA	CTX-M	Humans	*E. coli*	ST131, ST493, ST73, ST2346, ST1193, ST92, ST421, ST636, ST410, ST3268, ST120, ST602,	
				ST224, ST1196, ST2852, ST58, ST1011, ST6438,	
				ST167, ST8162, ST4981, ST1284, ST648, ST117, ST457, ST394, ST69, ST38, ST10	
			*K. pneumoniae*		
			*C. freundii*		
			*Enterobacter* spp.		
			*A. baumannii*	ST195	
		Community environment	*E. cloacae*		
	TEM	Humans	*E. coli*	ST493, ST73, ST131, ST2346, ST1193, ST92, ST421, ST636, ST410, ST4040, ST46, ST38, ST3268, ST405	
				ST648, ST117, ST394, ST69, ST8162, ST4981, ST1284	
			*A. baumannii*	ST195	
		Animals	*E. coli*		
	SHV	Humans	*E. coli*		
			*K. pneumoniae*		
			*C. freundii*		
			*Enterobacter* spp.		
		Animals	*E. coli*		
	OXA	Humans	*E. coli*		
			*P. aeruginosa*		
	GES	Humans	*A. baumannii*	ST1, ST2, ST15, ST17, ST113, ST114, ST115, ST116	
			*P. aeruginosa*		
	VEB	Humans	*P. aeruginosa*		
Egypt	CTX-M	Humans	*E. coli*	ST405, ST68, ST131, ST648; Phylogroup A, B1, B2, D	
			*K. pneumoniae*		
			*Enterobacter* spp.		
			*Salmonella* spp.		
			*P. mirabilis*		
			*C. freundii*		
			*S. marcescens*		
			*A. baumannii*		
			*P. aeruginosa*		
		Animals	*E. coli*	ST131	IncFII
				D	
	TEM	Humans	*K. pneumoniae*		
			*A. baumannii*		
		Animals	*E. coli*	B1, C, D	
			*Salmonella* spp.		
	SHV	Humans	*E. coli*	ST68, Phylogroups A and D	
			*A. baumannii*		
			*P. aeruginosa*		
		Animals	*E. coli*	D	
	OXA	Animals	*E. coli*	C. B2	

*References are cited in the main text.*

In Bahrain, CTX-M-grp1 and CTX-M-grp9 with high resistance to ciprofloxacin, nitrofurantoin, and trimethoprim-sulfamethoxazole have been described as the predominant ESBL types detected in *E. coli* and *K. pneumoniae* clinical strains (Bindayna and Murtadha, 2011; Shahid et al., 2014; Zowawi et al., 2014). In Qatar, CTX-M-group1, CTX-M-group9, TEM and SHV dominated the *E. coli* and *K. pneumoniae* clinical isolates (Sid Ahmed S.S. et al., 2016; Eltai et al., 2018b). In United Arab Emirates, CTX-M-15 and SHV-258 were detected in *K. pneumoniae* isolated from inpatients (Alfaresi et al., 2011). In parallel, CTX-M-15, CTX-M-3, and CTX-M-14 producing ST131 *E. coli* strains were also reported (Peirano et al., 2014). This same *E. coli* sequence type was isolated recently from the urine sample of a 76-year-old male patient. This isolate harbored the blaCTX-M-27 gene carried on an IncFII-FIA-FIB plasmid along with aminoglycosides (aadA5, strA, and strB), sulfonamide (sul1 and sul2), TET [tet(A)], macrolides (mphA), and trimethoprim (dfrA17), resistance determinants (Mutti et al., 2018). Ranjan Dash et al. (2018) found that age, gender, recurrent UTI and catheterization are significant factors for developing an ESBL UTI in United Arab Emirates.

In the Sultanate of Oman, the main risk factors for ESBL infections in children was suggested to include being female, severe illness, prolonged hospital stays and previous exposure to antimicrobials (Al Muharrmi et al., 2008). As for the ESBL types detected, only one study showed the presence of CTX-M-15 producing a clinical *E. coli* strain (Zowawi et al., 2014).

Last, but not least in the gulf region, in Yemen, CTX-M-15, SHV-11, SHV-76, and SHV-184 were detected in clonally diverse *K. pneumoniae* clinical isolates (Gharout-Sait et al., 2014). On the other hand, CTX-M-15 was observed in ST131 *E. coli* strains (Alsharapy et al., 2018). As it becomes evident, ST131 is highly associated with ESBL production in the Middle Eastern region as well as other countries across the world: Israel (Karfunkel et al., 2013), KSA (Alghoribi et al., 2015; Alyamani et al., 2017; Yasir et al., 2018), Iran (Moghanni et al., 2018), Bulgaria (Markovska et al., 2012), Ecuador (Zurita et al., 2019), and Spain (Merino et al., 2016).

In Egypt, ESBL producers are widely spread in hospitals. One recent study showed a significant association between 3rd generation cephalosporins and resistance fluoroquinolones, gentamicin and tetracycline in hospital acquired infections (Galal et al., 2018). CTX-M-1, CTX-M-9, CTX-M-15, CTX-M-14, and SHV-12 were reported in *E. coli* strains isolated from different clinical origins (Hassan et al., 2012; Abdelaziz et al., 2013a; Abdallah et al., 2015b; Helmy and Kashef, 2017).

Additionally, TEM and SHV variants were also reported by El-Badawy et al. (2017), who found that among 61 clinical isolates of *E. coli* producing ESBL, SHV-11, and TEM-214 were predominant followed by others such as SHV-48, TEM-206, TEM-57, TEM-135, TEM-207, TEM-34, and TEM-176. This study was the first to report the detection of GES in *E. coli* strains isolated from Egyptian patients. A total of 92.30% ESBL *E. coli* isolates belonged to the ST131 clone and 45.83% of them belonged to the O25b serotype (El-Badawy et al., 2017). The association of *E. coli* ST131 with high antimicrobial resistance and virulence was previously reported in the literature (Can et al., 2015). On the other hand, CTX-M-15, CTX-M-14, SHV-11, and SHV-12 were detected in ESBL positive *K. pneumoniae* strains (Abdelaziz et al., 2013a; Abdallah et al., 2015b). Considerable resistance against aminoglycosides, fluoroquinolones and trimethoprim-sulfamethoxazole was observed in these isolates (Abdallah et al., 2017). Other ESBL producers detected in Egyptian hospitals include CTX-M-14/CTX-M-15 *Enterobacter* spp. (Abdallah et al., 2015b; Galal et al., 2018), CTX-M-15/SHV *P. mirabilis*, CTX-M-15/SHV *C. freundii*, CTX-M-14 *Serratia marcescens* (Helmy and Kashef, 2017; Galal et al., 2018) and CTX-M *Salmonella* spp. (Abdallah et al., 2017). CTX-M-14, CTX-M-15, and SHV ESBL types were detected in *P. aeruginosa* and *A. baumannii* (Abdelkader et al., 2017; Alkasaby and El Sayed Zaki, 2017; Helmy and Kashef, 2017). Furthermore, as for AmpC production, CMY variants (CMY-2, CMY-42, CMY-102), DHA-1, EBC, FOX, and MOX were detected in clinical isolates of Enterobacteriaceae such as *E. coli*, *K. pneumoniae*, and *P. mirabilis* (Abdelaziz et al., 2013a; Helmy and Wasfi, 2014; Wassef et al., 2015).

### Infections With Carbapenemase Producers

In Iran, *K. pneumoniae* is the most common carbapenemase producing Enterobacteriaceae clinical isolate. Carbapenem resistance in these is often mediated by NDM-1 and OXA-48 production followed by KPC (KPC-2), VIM (VIM-1 and VIM-4), and IMP (Rastegar Lari et al., 2013; Azimi et al., 2014; Nobari et al., 2014; Fazeli et al., 2015; Rajabnia et al., 2015; Firoozeh et al., 2016, 2017; Sedighi et al., 2017; Shahcheraghi et al., 2017; Armin et al., 2018; Ghotaslou et al., 2018; Hosseinzadeh et al., 2018; Moghadampour et al., 2018a). The majority of the studies reported no clonal relatedness among isolated carbapenem resistant *K. pneumoniae* in each center (Shahcheraghi et al., 2013; Jafari et al., 2018; Kiaei et al., 2018). This is with the exception of three centers where an identical genotype was observed for VIM-1 producers (Nobari et al., 2014), for NDM-1 producers the strains were distributed into two major clonal complexes including ST13 and ST392 (Shoja et al., 2018) and among NDM-1 and/or OXA-48 positive ones the predominant cluster/pulsotype was associated to ST11 and ST893 (Solgi et al., 2018). In this latter study, OXA-48 and NDM-1 genes were located on IncL/M and IncFII plasmids, respectively. These transferable plasmids are known as potent contributors to the dissemination of resistance genes including NDM-1, OXA-48, and CTX-M-15 among enterobacterial species (Solgi et al., 2018). OXA-48 and NDM (NDM-1, NDM-7) were dominant in carbapenem resistant *E. coli* strains (Hojabri et al., 2017; Solgi et al., 2017b). In one study, isolated strains belonged to the ST131 (Hojabri et al., 2017) whereas in the other one, carbapenemase producing *E. coli* strains were distributed into 18 different sequence types with the predominance of ST648 and ST167 (Solgi et al., 2017b). Interestingly, in one of the aforementioned studies, OXA-48 was found on the same transferable plasmid type IncL/M that was previously detected in *K. pneumoniae* (Solgi et al., 2017b) (Table 2). This finding emphasizes the role of the IncL/M incompatibility group in the horizontal gene transfer of the OXA-48 gene among Enterobacteriaceae. In contrast, in the same study, NDM-1 was detected on an IncA/C plasmid type. In this study, ST648 and ST167 were dominant in NDM-1 and/or OXA-48 producing *E. coli* strains (Solgi et al., 2017b). Concerning *Salmonella*, two VIM positive strains were reported in Iran (Shahcheraghi et al., 2017). In *A. baumannii*, OXA-23 were dominant in all studies addressing carbapenem resistant *Acinetobacter* spp. in clinical settings (Azizi et al., 2015; Zanganeh and Eftekhar, 2015; Shoja et al., 2016, 2017; Mohajeri et al., 2017; Sarikhani et al., 2017; Zafari et al., 2017; Rezaei et al., 2018; Shirmohammadlou et al., 2018). Other carbapenem resistance genes included OXA-24, OXA-58, IMP, VIM, KPC, GIM, SIM, and SPM (Peymani et al., 2011; Azimi et al., 2015; Bagheri Josheghani et al., 2015; Aghamiri et al., 2016; Maspi et al., 2016; Moghadam et al., 2016; Khorvash et al., 2017; Armin et al., 2018; Soltani et al., 2018). Isolated strains of carbapenem resistant *A. baumannii* are genetically diverse with the predominance of International clone I and II (Peymani et al., 2011; Savari et al., 2017; Mahdian et al., 2015; Sarhaddi et al., 2017). The rapid evolution of bacterial resistance in *Acinetobacter* spp. could be attributed to its genome plasticity that allows the acquisition and loss of mobile genetic elements (plasmids, transposons) that modifies the organism’s genomic structure (Savari et al., 2017). As for non *baumannii* species, only one study reported the detection of OXA-23 and SPM producing *Acinetobacter nosocomialis* in patients with blood infections (Pourabbas et al., 2016). In *P. aeruginosa*, MBLs were the most common carbapenemases including: IMP (IMP-1 and IMP-55) and VIM variants (VIM-1, VIM-2) (Abiri et al., 2015; Lari et al., 2015; Mirbagheri et al., 2015; Moosavian and Rahimzadeh, 2015; Azizi et al., 2016; Saffari et al., 2016; Kazeminezhad et al., 2017; Dogonchi et al., 2018; Pournajaf et al., 2018; Rostami et al., 2018). Only two studies reported the detection of OXA-23 and SPM-1 in Iranian clinical isolates of *P. aeruginosa* (Ostad Asadolah-Malayeri et al., 2016; Azimi et al., 2018). Akhi et al. (2018) found that the main risk factor for acquiring an MBL infection is non-intensive wards hospitalization. Whether the dissemination of carbapenem resistant *P. aeruginosa* in Iran is polyclonal or not, cannot be assumed. This is because the genetic relatedness was investigated in only two studies; in one of these different genotypes (Akhi et al., 2018) were detected whereas in the other, the strains were distributed into three distinct genotypes (Azimi et al., 2018).

**TABLE 2:** Sequence and plasmid types associated with carbapenemase genes in humans, animals, and environment in the Middle East.

**Country**	**Carbapenemase gene**	**Reservoir**	**Species**	**Sequence type/phylogroup**	**Plasmid type**
Iran	Class D oxacillinases	Humans	*E. coli*	ST131, ST167, ST1431, ST5005, ST889, ST10, ST3737, ST410, ST5114, ST5164, ST315,	
				ST6350, ST648, ST167, ST410, ST431, ST3134	IncL/M
				ST178, ST6826	IncA/C
			*K. pneumoniae*	A, B	
			*A. baumannii*	ST2	
			*P. aeruginosa*		
		Hospital environment	*K. pneumoniae*		
			*A. baumannii*		
	KPC	Humans	*K. pneumoniae*	B	
	NDM	Humans	*E. coli*	C,D, ST167, ST131	
				ST3044, ST167, ST178, ST6826	IncA/C
				ST315	IncL/M
			*K. pneumoniae*	C, D	
				ST11, ST893	IncFII
			*Enterobacter* spp.		
			*A. baumannii*		
			*P. aeruginosa*		
		Community environment	*P. aeruginosa*		
		Hospital environment	*K. pneumoniae*		
	VIM	Humans	*K. pneumoniae*	ST13, ST392	
			*Salmonella* spp.		
			*A. baumannii*		
			*P. aeruginosa*		
		Community environment	*P. aeruginosa*		
	IMP	Humans	*K. pneumoniae*		
			*A. baumannii*		
			*P. aeruginosa*		
		Community environment	*P. aeruginosa*		
		Hospital environment	*K. pneumoniae*		
Turkey	Class D oxacillinases	Humans	*E. coli*		
			*K. pneumoniae*	ST11, ST258, ST16	
			*K. oxytoca*		
			*E. cloacae*		
			*E. aerogenes*		
			*P. mirabilis*		
			*P. stuartii*		
			*P. rettgeri*		
			*C. freundii*		
			*S. marcescens*		
			*M. morganii*		
			*R. planticola*		
			*A. baumannii*	ST2, ST15, ST157, ST10, ST158	
			*P. aeruginosa*		
	NDM	Humans	*E. coli*		
			*K. pneumoniae*		
			*E. cloacae*		
			*P. rettgeri*		
	VIM	Humans	*E. coli*		
			*K. pneumoniae*		
			*E. cloacae*		
			*P. aeruginosa*		
	IMP	Humans	*K. pneumoniae*		
			*E. cloacae*		
			*P. aeruginosa*		
Palestine/Israel	Class D oxacillinases	Humans	*P. mirabilis*		IncL/M
	KPC	Humans	*E. coli*	ST512	
			*K. pneumoniae*	ST340, ST277, ST376	
				ST327, ST512, ST258	pKpQIL
		Hospital and community environment	Enterobacteriaceae		
	NDM	Humans	*A. baumannii*	ST103, ST2, ST183	
		Hospital and community environment	Enterobacteriaceae		
	VIM	Humans	*P. aeruginosa*	ST1562, ST1563, ST1564	
Lebanon	Class D oxacillinases	Humans	*E. coli*	ST405	IncL/M
			*K. pneumoniae*		IncL/M
			*E. cloacae*		IncL/M
			*S. marcescens*		IncL/M
			*M. morganii*		IncL/M
			*R. ornithinolytica*		
			*A. baumannii*	ST2, ST25, ST1, ST570, ST85, ST600	
			*A. pittii*		
		Animals	*E. coli*	ST38	
			*A. baumannii*	ST2, ST491, ST493, ST20, ST492	
		Community environment	*E. coli*	ST38	IncHI2
				–	IncL
			*K. pneumoniae*	ST16	IncL
			*A. baumannii*		
	NDM	Humans	*K. pneumoniae*	ST14, ST25	
		Hospital environment	*E. cloacae*		
	VIM	Humans	*P. aeruginosa*	ST233, ST699, ST654	
		Animals	*P. aeruginosa*	ST1759, ST1760, ST1761, ST1762	
	IMP	Humans	*P. aeruginosa*	ST446, ST654	
Syria	Class D oxacillinases	Humans	*E. coli*	ST38	
			*K. pneumoniae*	ST101, ST1633	
	NDM	Humans	*E. coli*		
			*K. pneumoniae*	ST278	
			*E. cloacae*		
			*P. rettgeri*		
			*C. braakii*		
			*A. baumannii*	ST85	
Jordan	Class D oxacillinase	Humans	*K. pneumoniae*	A1, A3, B	IncL/M
		Hospital environment	*A. baumannii*		
	NDM	Humans	*E. coli*		
			*K. pneumoniae*	A2, C	
					IncFIB, IncFII
			*E. cloacae*		
	VIM	Humans	*E. cloacae*		
Iraq	Class D oxacillinases	Humans	*A. baumannii*	ST2, ST136, ST194, ST623, ST792, ST793	
	NDM	Humans	*K. pneumoniae*		
			*P. aeruginosa*		
	IMP	Humans	*K. pneumoniae*		
			*P. aeruginosa*		
Kuwait	Class D oxacillinases	Humans	*E. coli*	ST405, ST38	
			*K. pneumoniae*	ST677, ST16, ST107, ST485	
			*E. cloacae*		
			*M. morganii*		
			*A. baumannii*	ST158	pK50a
				ST195, ST208, ST229, ST436, ST450, ST452, ST499	
	NDM	Humans	*E. coli*	ST448	IncX3
			*K. pneumoniae*		
			*E. cloacae*		
			*P. stuartii*		
			*M. morganii*		
	VIM	Humans	*E. coli*		IncA/C
			*K. pneumoniae*		IncA/C
			*K. oxytoca*		
			*E. cloacae*		
			*P. aeruginosa*		
	IMP	Humans	*A. baumannii*		
KSA	Class D oxacillinases	Humans	*E. coli*		
			*K. pneumoniae*	ST29, ST709, ST37, ST111, ST974, ST29, ST199, ST48, ST348, ST152, ST340, ST17, ST298, ST16,	
				ST11, ST353	
			*E. cloacae*		
			*E. aerogenes*		
			*A. baumannii*	ST2, ST19, ST20, ST25, ST236, ST1096, ST945, ST208,	
				ST450, ST452, ST499, ST195, ST229, ST436,	
	NDM	Humans	*K. pneumoniae*	ST152, ST348, ST199, ST1045	
			*E. cloacae*		
			*E. aerogenes*		
			*A. baumannii*		
		Community environment	*E. coli*		
	VIM	Humans	*K. pneumoniae*		
			*K. oxytoca*		
			*E. cloacae*		
			*A. baumannii*	ST1, ST2, ST195, ST196, ST487, ST489, ST20	
	IMP	Humans	*A. baumannii*	ST1, ST2, ST195, ST196, ST487, ST489, ST20	
Bahrain	Class D oxacillinases	Humans	*A. baumannii*	ST208, ST452	
	VIM	Humans	*P. aeruginosa*		
Qatar	Class D oxacillinases	Humans	*E. coli*		
			*K. pneumoniae*		
			*A. baumannii*	ST229	
	NDM	Humans	*K. pneumoniae*		
	VIM	Humans	*P. aeruginosa*		
United Arab Emirates	Class D oxacillinases	Humans	*E. coli*		
			*K. pneumoniae*	ST14, ST15, ST23, ST48	
			*Citrobacter* spp.		
			*S. marcescens*		
	KPC	Humans	*K. pneumoniae*	ST14	IncX3
	NDM	Humans	*E. coli*		
			*K. pneumoniae*	ST14	HI1b
				ST147	IncFII
			*E. cloacae*		
	VIM	Humans	*K. pneumoniae*		
Oman	Class D oxacillinases	Humans	*E. coli*	ST648	IncL/M
				ST138	
			*K. pneumoniae*	ST753, ST754	IncL/M
			*A. baumannii*	ST195	
	NDM	Humans	*E. coli*	ST2527	IncF
				ST4107	IncX3
			*K. pneumoniae*	ST15, ST147	IncH1B
				ST372	IncFII
				ST11	IncL/M, IncH1B
	VIM	Humans	*K. pneumoniae*		
Yemen	Class D Oxacillinase	Humans	*A. baumannii*	ST2	
	NDM	Humans	*K. pneumoniae*	ST1399, ST147, ST29, ST405, ST340	
			*E. cloacae*		
Egypt	Class D oxacillinases	Humans	*E. coli*	ST101, ST648	
			*K. pneumoniae*		
			*Salmonella* spp.		
			*S. marcescens*		
			*A. baumannii*	ST331, ST410, ST208, ST108, ST411, ST409, ST236	
			*P. aeruginosa*		
		Animals	*K. pneumoniae*		
	KPC	Humans	*K. pneumoniae*		
			*E. cloacae*		
			*A. baumannii*		
			*P. aeruginosa*		
		Animals	*K. pneumoniae*		
	NDM	Humans	*E. coli*	ST5018	Incl1-ly
			*K. pneumoniae*	ST147, ST11, ST17	IncR
			*E. cloacae*		
			*S. marcescens*		
			*A. baumannii*	ST103	
			*P. aeruginosa*	ST233	
		Animals	*K. pneumoniae*		
	VIM	Humans	*E. coli*	A, B	
			*K. pneumoniae*		
			*S. maltophilia*		
			*A. baumannii*		
			*P. aeruginosa*	ST233, ST198, ST303, ST629, ST507, ST406, ST274, ST990, ST683, ST884, ST738	

*References are cited in the main text.*

In Turkey, *K. pneumoniae* were the most common GNB resistant to carbapenems (Us et al., 2010). Resistance to these agents is mainly mediated via the production of OXA-48 carbapenemases (Nazik et al., 2012, 2014; Iraz et al., 2015; Baran and Aksu, 2016; Haciseyitoglu et al., 2017; Kutlu et al., 2018). Some studies found a clonal relationship between isolated OXA-48 strains in the clinical center investigated (Castanheira et al., 2014; Karabay et al., 2016; Haciseyitoglu et al., 2017) while others did not (Kilic et al., 2011; Nazik et al., 2012; Iraz et al., 2015). Interestingly, in one of the studies, post the detection of a clonal spread of OXA-48 *K. pneumoniae* in a tertiary care center, infection control measures including hand washing, high level surface disinfection, screening of colonization before admission were strictly followed (Ece et al., 2018). Later on, this resulted in a significant decrease of the rate of carbapenem resistant *K. pneumoniae* (Ece et al., 2018). Other types of carbapenemases were also detected in Turkish isolates of *K. pneumoniae*: NDM, VIM and IMP (Balkan et al., 2014; Candan and Aksoz, 2015; Cakar et al., 2016; Guven Gokmen et al., 2016). In *E. coli* strains, OXA-48, NDM, and VIM were detected (Gulmez et al., 2008; Carrer et al., 2010; Kilic et al., 2011; Nazik et al., 2012; Baron et al., 2016; Cakar et al., 2016; Kuskucu et al., 2016; Kutlu et al., 2018). Other carbapenemases producing Enterobacteriaceae include: OXA-48 and MBL *Enterobacter* species (Carrer et al., 2010; Baron et al., 2016; Haciseyitoglu et al., 2017), OXA-48 *C. freundii*, *S. marcescens*, *P. mirabilis*, *M. morganii*, *Raoultella planticola*, *P. stuartii*, and *Providencia rettgeri* (Carrer et al., 2010; Baron et al., 2016). Otlu et al. (2018) reported the detection of two genetically indistinguishable OXA-48/NDM-1 producing *P. rettgeri* isolates. These strains were isolated from two different patients about 7 months apart in the same unit. These data show that Turkey is an endemic area of OXA-48 producers, thus warranting the urgent implementation of infection control measures as well as antibiotic stewardship programs (ASP). In non-fermenters, OXA-23, OXA-24, and OXA-58 were detected in *A. baumannii* with no evidence of clonal dissemination (Kulah et al., 2010; Ciftci et al., 2013; Metan et al., 2013; Castanheira et al., 2014; Cicek et al., 2014; Aksoy et al., 2015; Ahmed S.S. et al., 2016; Direkel et al., 2016). In two studies, co-resistance to colistin was detected (Ergin et al., 2013; Keskin et al., 2014); this fact is threatening and should be taken into real consideration since colistin is currently a last resort therapeutic agent against carbapenem resistant organisms (Olaitan et al., 2014b). On the other hand, MBL (VIM-1, VIM-2, VIM-38, IMP-1, and IMP-9) (Iraz et al., 2014; Yilmaz et al., 2014; Er et al., 2015; Malkocoglu et al., 2017) and OXA-23/OXA-58 were detected in clonaly diverse *P. aeruginosa* (Tasbent and Ozdemir, 2015).

In Syria, the epidemiology of MDR is unknown due to the civil war crisis. However, injured Syrian refuges are considered a source of MDROs in the country they are residing in Peretz et al. (2014b), Reinheimer et al. (2016). Indeed, recent studies showed the introduction of ST85 NDM-1 positive *A. baumannii* into Lebanese clinical settings from wounded Syrian refugees (Rafei et al., 2014b, 2015b). Subsequently, NDM-1 positive *Acinetobacter* spp. were isolated from Lebanese patients (Rafei et al., 2015b). Similarly, NDM/OXA-48 *K. pneumoniae* and *E. coli* and NDM producing *E. cloacae*, *P. rettgeri*, and *Citrobacter braakii* were isolated from Syrian refugees in North Palestine (Lerner et al., 2016). In both reports, the origin of the detected isolates could not be determined; the infection might have been acquired on the battlefield from environmental sources, during the patients stay in Syrian hospitals or during evacuation from Syria to another territory (Rafei et al., 2014b). Instead, what is sure is that screening of refugees arriving from countries with unknown epidemiology of carbapenem resistance, upon hospital admission, is a must and is crucial in order to contain the dissemination of these highly resistant MDROs (Lerner et al., 2016).

In Lebanon, in early 2012, OXA-48 and OXA-48/NDM-1 positive *E. coli* and *K. pneumoniae*, respectively, were isolated from the blood and urine cultures of Iraqi patients (El-Herte et al., 2012). Indeed, the most common carbapenemases detected in Enterobacteriaceae isolated from Lebanese hospitals are class D oxacillinases; these include OXA-48/OXA-232 *E. coli*, ST14 NDM-1 *K. pneumoniae*, OXA-48, OXA-162, OXA-232 *K. pneumoniae*, OXA-48/OXA-232 *E. cloacae* and OXA-48 producing *S. marcescens*, *M. morganii*, and *Raoultella ornithinolytica* (Hammoudi et al., 2015b; Al-Bayssari et al., 2016; Tokajian et al., 2016; Hammoudi Halat et al., 2017; Alousi et al., 2018). In two studies, the OXA-48 gene was located on the same plasmid IncL/M in *E. coli* and *K. pneumoniae* alike (Hammoudi Halat et al., 2017; Alousi et al., 2018). This emphasizes the crucial role that mobile genetic elements play in the spread of resistance determinants between different species. On the other hand, within the same species, no clonal relatedness was observed among carbapenem resistant *K. pneumoniae* strains (Baroud et al., 2013). NDM-1 producing *K. pneumoniae* belonging to ST14 was reported by Alousi et al. (2018). Furthermore, an NDM-1 ST15 *K. pneumoniae* strain was isolated from the urine sample of an old Syrian refugee in Lebanon (Salloum et al., 2017). ST15 is heavily reported in hospitals worldwide such as Nepal (Stoesser et al., 2014), Vietnam (Tada et al., 2017), Thailand (Netikul et al., 2014), and China (Hu et al., 2013). The successful dissemination of ST15 *K. pneumoniae* could be attributed to its ability to acquire several resistance genes with no fitness cost (Toth et al., 2014). On the other hand, bacterial resistance in *A. baumannii* have largely evolved in Lebanon since its first detection by Matar et al. (1992). OXA-58 were at first the most common carbapenemase detected in clinical isolates of *A. baumannii* (Giannouli et al., 2009; Di Popolo et al., 2011), thereafter, OXA-23 and OXA-24 dominated (Rafei et al., 2014a; Hammoudi et al., 2015a, b; Hammoudi Halat et al., 2017). The dissemination of carbapenem resistant *A. baumannii* in Lebanese hospitals appears to be mainly mediated via the international clone II (Al Atrouni et al., 2016; Dahdouh et al., 2016; Hajjar Soudeiha et al., 2018). However, horizontal gene transfer has also played a major role. This is illustrated in a study conducted by Kanj et al. (2018) who found that the prevalence of OXA-23 positive *A. baumannii* have significantly increased between 2007, 2008, and 2013. Molecular analysis revealed only a 22% genomic relatedness among isolated strains. This emphasizes the role of horizontal gene transfer in the dissemination of resistance determinants among *A. baumannii* in Lebanese hospitals. Only one study reported the detection of non *baumannii Acinetobacter* species: *Acinetobacter pittii* producing NDM-1 and OXA-72 carbapenemases; these strains were isolated from the urine culture of a 4-month-old child and from a febrile gastroenteritis infected patient, respectively (Al Atrouni et al., 2016a). As for *P. aeruginosa* isolated from Lebanese hospitals, only MBLs were detected: IMP-1, IMP-2, IMP-15, and VIM-2 (Al Bayssari et al., 2014; Hammoudi et al., 2015b; Hammoudi Halat et al., 2017).

In Israel, KPC-3 and to a lesser extent KPC-2 producing *K. pneumoniae* appear to be endemic (Navon-Venezia et al., 2009; Leavitt et al., 2010a, b; Warburg et al., 2012; Castanheira et al., 2014). PFGE analysis showed that isolated KPC-3 *K. pneumoniae* belonged to the same genetic clone (Navon-Venezia et al., 2009). On the other hand, MLST analysis in three other studies showed the predominance of ST258 (Leavitt et al., 2010a; Warburg et al., 2012; Castanheira et al., 2014). The fact that KPC was also detected in other enterobacterial species such as *E. coli* (KPC-2) (Goren et al., 2010a, b), *Enterobacter* spp. (Lazarovitch et al., 2015), and *C. freundii* (Castanheira et al., 2014) suggests a possible monoclonal spread of KPC in *K. pneumoniae* and its subsequent successful horizontal gene transfer to other species. Other carbapenemase producing GNB detected in Palestine involve: OXA-48 located in the IncL/M plasmid in *P. mirabilis* (Chen et al., 2015), VIM-2/VIM-4 *P. aeruginosa* in Palestine (Sjolander et al., 2014) and NDM-2/OXA-23/OXA-24 *A. baumannii* in both Israel and Palestine (Castanheira et al., 2014; Sjolander et al., 2014).

In Jordan, NDM-1 and NDM-1/VIM-4 were detected in *E. coli* and *E. cloacae* clinical isolates, respectively (Aqel et al., 2018). Furthermore, two studies reported the detection of NDM and OXA-48 in genetically diverse *K. pneumoniae* strains isolated from clinical specimens (Aqel et al., 2017, 2018). In one study, NDM-1 and OXA-48 were located on FII(K)/FIB and IncL/M, respectively (Aqel et al., 2018). The interesting finding in the second study is that distinct NDM-1 positive *K. pneumoniae* were isolated from a Yemeni patient and a native Jordanian without a history of travel, hospitalized at the same time period. In the same report, distinct OXA-48 *K. pneumoniae* was isolated from a Yemeni and also a native Jordanian treated in the same ward with specimens 12 days apart (Aqel et al., 2017). Altogether, these data highlight the importance of horizontal gene transfer and the absence of effective infection control measures in the dissemination of carbapenem resistance genes in Jordanian hospitals.

In Iraq, the most common carbapenemase producers are the non-fermenters including OXA-23/OXA-24 *A. baumannii* (Kusradze et al., 2011; Ganjo et al., 2016) and NDM (NDM-1, NDM-2), IMP and SPM *P. aeruginosa* (Al-Charrakh et al., 2016; Ismail and Mahmoud, 2018). According to Al-Charrakh et al. (2016), the high resistance of carbapenem resistant *P. aeruginosa* to non-beta lactams such as ciprofloxacin and gentamicin, can be attributed to the over-use of these antimicrobial agents in Iraqi clinical practices. In Enterobacteriaceae, only NDM-1 and SPM *K. pneumoniae* strains were detected (Hussein, 2018).

In Kuwait, VIM-4, NDM (NDM-1, NDM-7), and OXA-48 carbapenemases were detected in clinical isolates of clonally unrelated *E. coli* and *K. pneumoniae* strains (Jamal et al., 2013, 2015, 2016; Pal et al., 2017). In one study, the blaVIM gene was located on the same plasmid type IncA/C both in *E. coli* and *K. pneumoniae* (Sonnevend et al., 2017b). Moreover, several reports described the detection of NDM-1 *P. stuartii*, OXA-48, NDM-1 *M. morganii* and VIM (VIM-4), NDM-1, and OXA-48 *E. cloacae* in Kuwaiti hospitals (Jamal et al., 2013, 2015, 2016; Sonnevend et al., 2015a, 2017b). In *A. baumannii*, OXA-23 were mainly detected followed by IMP-1, VIM (VIM-1 and VIM-2) (Jamal et al., 2009; Al-Sweih et al., 2012; Zowawi et al., 2015; Wibberg et al., 2018). On the other hand, only one study reported the detection of VIM positive *P. aeruginosa* clinical strains (Zowawi et al., 2018). The *P. aeruginosa* strains were distributed into 14 sequence type clusters with some of them being recognized as highly disseminated international clones such as ST111, ST235, ST357, and ST654 (Zowawi et al., 2018). In fact, according to one report, it has been suggested that the dissemination of carbapenem resistance in the clinical settings of Kuwait appears to be promoted by immigration, in-sufficient infection control measures, environmental spread, and antibiotic misuse (Jamal et al., 2016).

In KSA, OXA-48, and MBL (NDM-1, VIM-4, and VIM-29) are the most common carbapenem resistance genes detected in Enterobacteriaceae (Memish et al., 2015; Sonnevend et al., 2015b; Algowaihi et al., 2016; Alotaibi et al., 2017; Al-Zahrani and Alsiri, 2018; Zaman et al., 2018). These isolates included mainly *K. pneumoniae* and others (*E. coli*, *E. cloacae*, and *Enterobacter aerogenes*) (Al-Agamy et al., 2013; Shibl et al., 2013; Uz Zaman et al., 2014; Memish et al., 2015; Abdalhamid et al., 2017b). In fact, several studies reported a clonal relatedness among carbapenemase producing *K. pneumoniae* in each clinical center (Balkhy et al., 2012; Uz Zaman et al., 2014; Abdalhamid et al., 2017b). In one study, the clonal relatedness of carbapenem resistant *K. pneumoniae* was 93.2% (Abdalhamid et al., 2017b) whereas in other studies, MLST analysis revealed the predominance of certain sequence types such as ST29, ST199, and ST152 (Uz Zaman et al., 2014; Zaman et al., 2018). Furthermore, in one of the reports, the IncL/M plasmid type was predominant in OXA-48 *Klebsiella* spp. (Zaman et al., 2018). Indeed, one explanation for the MBL and OXA-48 predominance in the clinical isolates of Enterobacteriaceae in KSA is the big number of migrant workers and visitors coming from endemic areas such as India, Pakistan and Turkey (Al-Zahrani and Alsiri, 2018). Moreover, one study found that most of the patients infected with a carbapenem resistant *K. pneumoniae* had prolonged hospital stays, indwelling devices, surgical procedures, carbapenem usage and infection/carriage with MDROs (Balkhy et al., 2012). On the other hand, class D oxacillinase (OXA-23, OXA-24, and OXA-58) predominate in *A. baumannii* followed by NDM, VIM, and IMP (Alsultan et al., 2013; Elabd et al., 2015; Aly et al., 2016; Al-Agamy et al., 2017; Alhaddad et al., 2018). Clonal diversity revealed by different sequence types as well as PFGE patterns among isolated strains was reported in all the studies (Aly et al., 2014; Lopes et al., 2015; Zowawi et al., 2015; El-Mahdy et al., 2017). In one study, Aljindan et al. (2015) found that carbapenem resistant *A. baumannii* were more resistant to gentamicin, amikacin, ciprofloxacin, and tigecycline compared to the susceptible ones. In *P. aeruginosa* with high clonal diversity, VIM, IMP, VIM-1, VIM-2, VIM-4, VIM-11, VIM-28 were detected (Al-Agamy et al., 2012, 2016; Tawfik et al., 2012).

In Bahrain, VIM and class D oxacillinases (OXA-23, OXA-58, OXA-72, and OXA-40) were detected in genetically variant *P. aeruginosa* and *A. baumannii*, respectively (Mugnier et al., 2009; Zowawi et al., 2015, 2018). In Qatar, OXA-48 *E. coli*, NDM/OXA-48 *K. pneumoniae*, OXA-23 *A. baumannii*, and VIM *P. aeruginosa* were reported in clinical settings (Zowawi et al., 2014, 2015, 2018; Rolain et al., 2016).

In United Arab Emirates, NDM (NDM-1 and NDM-5), OXA-48 and to a lesser degree KPC, are the predominant carbapenemases detected in clinical isolates of *K. pneumoniae* (Dash et al., 2014; Sonnevend et al., 2015a, 2017a). MLST analysis revealed different sequence types with the most common being ST11, ST14, and ST147 (Sonnevend et al., 2013, 2015a, 2017a; Moubareck et al., 2018). ST147 is of special interest since in their study, Sonnevend et al. (2017a) reported a multi-hospital occurrence of a pan-resistant ST147 *K. pneumoniae* isolated from four patients over a 1 year period. The strains had highly similar genotypes and PFGE patterns. Furthermore, with more deep genetic analysis, extensive similarities (backbone and resistance islands) were found between these strains and the ST147 *K. pneumoniae* strains isolated in South Korea. Interestingly, one of the Korean isolates was from a patient transferred from the United Arab Emirates. This reveals the huge capacity of the ST147 *K. pneumoniae* clone in maintaining itself over a long period of time in addition to its ability to be transmitted internationally (Sonnevend et al., 2017a). Similarly, NDM and OXA-48 were also found in other GNB in the Imarati hospitals including *E. coli*, *E. cloacae*, *Citrobacter* spp., *S. marcescens*, and *A. baumannii* (Ghazawi et al., 2012; Sonnevend et al., 2013, 2015b). In two studies, the NDM gene was located on an IncX3 plasmid (Sonnevend et al., 2013; Pal et al., 2017; Moubareck et al., 2018). According to Sonnevend et al. (2013), the Middle East is the second region where IncX3 plasmids with very similar structures that carry the blaNDM-1 were detected; found in different species, this emphasizes the role of this plasmid type on the inter-generic dissemination of this MBL gene.

In Oman, carbapenem resistance in Enterobacteriaceae (*E. coli* and *K. pneumoniae*) is mediated via the production of NDM (NDM-1 and NDM-7) and OXA-48 carbapenemases (Dortet et al., 2012; Zowawi et al., 2014). Reported sequence types for *K. pneumoniae* include ST14, ST340, ST11, and ST147 (Poirel et al., 2011a; Potron et al., 2011; Sonnevend et al., 2015a). Furthermore, as reported in United Arab Emirates, NDM-7 in *E. coli* was located on the epidemiologically important IncX3 plasmid (Pal et al., 2017). On the other hand, OXA-23 was detected in *A. baumannii* whereas in *P. aeruginosa* VIM and IMP were found (Zowawi et al., 2015, 2018). In the gulf, Zowawi et al. (2015) found that several clusters of indistinguishable OXA-23 *A. baumannii* strains are circulating. These include ST208 and ST195 that belong to the clonal complex 92, which is internationally disseminated (Chen et al., 2017; Rieber et al., 2017).

In Yemen, OXA-23 producing ST2 *A. baumannii* were isolated from clinical settings (Bakour et al., 2014); this is in addition to clonally un-related NDM-1 *K. pneumoniae* (ST1399, ST147, ST29, ST405, and ST340) and *E. cloacae* strains (Gharout-Sait et al., 2014).

In Egyptian hospitals, KPC, VIM (VIM-1, VIM-2, and VIM-29), NDM (NDM-1 and NDM-5), and OXA-48 are the predominant carbapenamases detected in Enterobacteriaceae (Abdelaziz et al., 2013b; Metwally et al., 2013; Hamdy Mohammed el et al., 2016; Abdallah et al., 2017; Barwa and Shaaban, 2017; Khalifa et al., 2017; Khalil et al., 2017; Abdulall et al., 2018; Kamel et al., 2018). Molecular analysis revealed that no clonal relationship was observed among carbapenem resistant *E. coli* and *K. pneumoniae* strains (Abdelaziz et al., 2013b; Khalifa et al., 2017; Khalil et al., 2017; Abdulall et al., 2018). The polyclonal spread of carbapenem resistant *K. pneumoniae* in Egypt is further documented in a study conducted in Italy. In this study, two NDM producing *K. pneumoniae* were isolated from unrelated patients with recent hospitalization in an Egyptian hospital (Principe et al., 2017). Isolated strains belonged to different sequence types. ST15 which was previously reported in Africa (Poirel et al., 2011b) and other Middle Eastern countries such as Lebanon (Salloum et al., 2017); and ST11 which is the sequence type to which the first NDM-1 *K. pneumoniae* strain isolated from Egypt belonged to (Abdelaziz et al., 2013b; Gamal et al., 2016). Polyclonal and horizontal gene transfer via mobile genetic elements appears to play an important role in the spread of carbapenemase producers in Egyptian clinical settings. However, more genetic analyses (MLST, plasmid typing) are needed to confirm this assumption. Other carbapenemase producers detected in Egyptian clinical settings include: VIM, KPC, NDM *E. cloacae*, OXA-48 *M. morganii* and *Salmonella*, OXA-48, NDM-1 *S. marcescens* and VIM *Stenotrophomonas maltophilia* (Hamdy Mohammed el et al., 2016; Khalifa et al., 2017; Abdulall et al., 2018; Kamel et al., 2018). In non-fermenters, carbapenem resistance *A. baumannii* was mediated mainly via OXA-23, OXA-24, OXA-58 followed by NDM (NDM-1 and NDM-2), VIM (VIM-1 and VIM-2), IMP, SIM, and GIM (Kaase et al., 2011; Fouad et al., 2013; El-Ageery and Al-Hazmi, 2014; Lopes et al., 2015; Hamdy Mohammed el et al., 2016; Alkasaby and El Sayed Zaki, 2017; Ghaith et al., 2017; Abdulall et al., 2018; Abdulzahra et al., 2018; Kamel et al., 2018; Ramadan R.A. et al., 2018). High genetic diversity was observed among isolated strains (Al-Hassan et al., 2013; Ghaith et al., 2017; El Bannah et al., 2018). As for associated risk factors, one study showed that the empirical intake of carbapenem 1 month ago is significantly associated with the development of a carbapenem resistance caused infection (ElMahallawy et al., 2018). On the other hand, VIM (VIM-2, VIM-28, and VIM-1-like), NDM (NDM-1), IMP, and OXA-48 genes were reported in *P. aeruginosa* (El-Mahdy, 2014; Zafer et al., 2014, 2015; Khalifa et al., 2017). The majority of isolated strains were genetically diverse with different sequence types including ST233, ST303, ST198, ST629, and ST507 (Zafer et al., 2014, 2015; Khalifa et al., 2017).

### Infections With Colistin Resistant Gram-Negative Bacilli

In Egypt, the first *mcr*-1 producing *E. coli* isolated from a clinical setting occurred in 2016. This strain co-produced the CTX-M-15 and had a sequence type of ST1011 which was previously detected in an avian *E. coli* strain within this same country. This finding could be a direct manifestation of a zoonotic transmission of *mcr*-1 from animals to humans (Elnahriry et al., 2016). Another study conducted on carbapenem resistant *A. baumannii* revealed substitutional mutations in the pmrA/B genes and subsequent colistin resistance. *A. baumannii* is considered an opportunistic pathogen and is usually treated with colistin if found to be carbapenem resistant. This association of colistin resistance with resistance to other antimicrobials is thus especially worrisome (Abdulzahra et al., 2018).

In Lebanon, Okdah et al. (2017) reported the detection of colistin resistance in three unrelated *K. pneumoniae* strains (ST268, ST2296, and ST348) with mutations in the mgrB, phoQ, pmrA/B genes in a hospital in Beirut. In Israel, one study reported the case of an Israeli patient with prior colistin administration during hospitalization and subsequent isolation of colistin resistant *K. pneumoniae* from his stool, supporting the theory of colistin resistance emergence as a result of antibiotic overuse in hospitals (Olaitan et al., 2014a). Lalaoui et al. (2019), reported the detection of colistin resistance in NDM-1 and KPC-3 harboring *K. pneumoniae* strains isolated from a medical center in Jerusalem. Resistance to colistin in these isolates was mediated by inactivation of the mgrB gene via an IS5-like insertion sequence (Lalaoui et al., 2019). Similarly, to nearby countries, colistin resistant *K. pneumoniae* strains in Israel are genetically diverse with different sequence types including ST16, ST76, ST258, and ST512 (Lalaoui et al., 2019). Indeed, this country lacks quantitative investigation of the dosages and/or duration of colistin administration that significantly increase the risk of development of colistin resistance in a strain or a patient (Drozdinsky et al., 2018).

In Jordan, Nazer et al. (2015) conducted a study where they focused on critically ill cancer patients with carbapenem resistant *A. baumannii* and the adverse effects of colistin as choice of treatment. In the latter, despite 66% of the patients being cleared of their respiratory infections with colistin resistant *A. baumannii*, nephrotoxicity and even mortality were significantly associated with this therapeutic regimen. This warrants quantitative studies that are not necessarily targeted at determining doses and frequency that lead to emergence of *A. baumannii* colistin resistant strains, but are rather targeted at finding treatments for different types of infections in different populations (critically ill cancer patients for example) with minimal side effects and optimal outcomes (Nazer et al., 2015).

In the region of the Arabian Peninsula, colistin resistance is a public health challenge that is worth addressing. In the United Arab Emirates for instance, *K. pneumoniae* strains were isolated from different hospitals in different emirates. ST147 *K. pneumoniae* was isolated from a hospital in Abu Dhabi as well as from two different hospitals in Um al Quwain (Sonnevend et al., 2017a). This strain was not only carbapenem resistant through the blaOXA-181 gene but was also colistin resistant through an insertion in its mgrB gene. Interestingly enough, the insertion into the mgrB gene which resulted in colistin resistance was in fact the functional blaOXA-181 gene (Sonnevend et al., 2017a). Those findings imply that not only is there a spread of this ST over a large geographic area, but also that this strain is one of many that have developed resistance to both carbapenems and colistin and therefore has the potential to cause epidemics (Sonnevend et al., 2017a). Moreover, a study conducted in Dubai, on clinical isolates from hospitals with the broadest medical and surgical exposure in the country to assess resistance to carbapenems as well as to colistin, found that 31.4% of the carbapenem resistant *K. pneumoniae* strains isolated were also colistin resistant (Moubareck et al., 2018). The mechanism of colistin resistance was not identified but was confirmed not to be the *mcr* plasmid mediated gene. While 40% of the *K. pneumoniae* isolates that were both colistin and carbapenem resistant were sporadic cases, 31.4% were associated with the *K. pneumoniae* ST14 clone, which is locally prevalent. Along with the fact that Dubai is a major economical, touristic, and medical city in the region, the above information showcases the potential of Dubai playing an important role in the spread of colistin resistance from a One Health Concept perspective (Moubareck et al., 2018). Indeed, in the UUnited Arab EmiratesAE, only one ST131 *E. coli* strain harboring the *mcr-1* gene was reported (Sonnevend et al., 2016).

In Qatar, a colistin resistant clinical *E. coli* strain positive for the *mcr-1* gene was recently reported. This isolate belongs to ST95, known to cause meningitis in humans as well as severe avian infections. It is worth mentioning that this strain had an ISApl1 element in the same plasmid carrying the *mcr-1* gene and the pap2-like phosphatase gene (Forde et al., 2018). The PAP2-like phosphatase can potentially contribute to colistin resistance by modifying the lipid A of the GNB outer membranes. The extent to which this gene contributes to colistin resistance in bacteria remains unknown but is worth investigating (Forde et al., 2018). Similarly, in Bahrain, the clinical colistin resistant *E. coli* strains (ST648 and ST224) were associated with the *mcr*-1 gene being on an Incl2 plasmid type (Sonnevend et al., 2016).

Additionally, in Oman, a clinical isolate of *E. coli* carrying *mcr*-1 was isolated in 2016. This strain belongs to ST10 and also harbors a plasmid of the IncI2 type. The detection of colistin resistance in ST10 *E. coli* is worrisome given that this clonal group has been known to mediate the spread of ESBL and quinolone resistance genes globally (Mohsin et al., 2018). In Kuwait, the development of colistin resistance in *Acinetobacter* spp. was evaluated in 2011. Of a total of 250 strains collected from eight governmental hospitals, 12% were found to be resistant to colistin. Compared to 0% in 2009, this significant increase prevalence could be attributed to the sudden increase in colistin prescription due to the global emergence of MDR infections (Al-Sweih et al., 2011).

In the KSA, a study by Mirnejad et al. (2018), focused on resistance to polymyxin B rather than colistin (polymyxin E). Those two antibiotics however, cover the same spectrum of organisms and can be used interchangeably as they have very similar mechanisms of action (Mirnejad et al., 2018). It was found that 13.2% of *A. baumannii* strains collected were resistant to polymyxin B (Memish et al., 2012). Another study found that the rate of resistance to colistin among *A. baumannii* in the KSA increased from 2.6 to 4.7% over the course of 2 years between 2010 and 2011 (Baadani et al., 2013). The danger that accompanies the appearance of colistin resistant strains in this country was embodied in a study in which two out of seven patients involved died due to colistin resistance. In that study, there was a history of colistin use reported in all patients except for one, suggesting that sporadic emergence rather than horizontal transmission of resistance might have played a more important role in the rise of colistin resistance in the isolated strains (Garbati et al., 2013). Moreover, sporadic cases of *mcr*-1 in hospitals in the KSA has previously been reported (Sonnevend et al., 2016). However, most of the studies conducted demonstrated chromosomal mutations (mgrB and phoP) responsible for colistin resistance (Uz Zaman et al., 2018). In all of these studies, no clonal relatedness was observed among isolated colistin GNB strains (Sonnevend et al., 2016; Uz Zaman et al., 2018). The polyclonal spread of colistin resistance questions the level of colistin use in hospitals of the Arabian peninsula.

While addressing the topic of colistin resistance and the One Health Concept in the KSA, it is very important to mention the yearly Muslim pilgrimage, Hajj, that takes place in the city of Mecca. Plasmid mediated *mcr*-1 carrying strains of predominantly genetically diverse *E. coli* strains and to a lesser extent *K. pneumoniae* have previously been isolated from patients during the Hajj. Pilgrims arrive from different countries, different occupations, and therefore with different sources of colistin resistance acquisition. These sources might be from the environment, food, animals, or from other humans (Leangapichart et al., 2016b).

In Turkey, colistin resistance has raised great concern as it has been associated with poor prognosis (Yilmaz et al., 2016). A study done by Cizmeci et al. (2017), found that six out of eight patients with *K. pneumoniae* that are resistant to both carbapenems and colistin ended up dying when all treatment options failed. Carbapenem resistant isolates positive for the NDM-1 gene have been found to have a higher rate of concomitant colistin resistance than isolates positive for the OXA-48 gene (Cizmeci et al., 2017). Furthermore, not only is the potential for colistin resistant infections to be fatal worrisome in Turkey, their potential to cause epidemics is also worrisome; the isolation of identical colistin resistant strains circulating in the country over short periods of time validate those concerns (Metan et al., 2017).

In Iran, one study reported the isolation of two colistin resistant *P. aeruginosa* from university teaching hospitals. The two isolates presented with different sequence types and more importantly were isolated from patients with no history of colistin consumption. The mechanisms of colistin resistance in both isolates was the overexpression of MexB and MexY genes which code for MexAB-OprM and MexXY-OprM efflux pumps. Despite colistin not being a specific substrate for those efflux pumps, the over expression of the MexAB-OprM and MexXY-OprM efflux was suspected to have played a role in the development of colistin resistance (Goli et al., 2016). This theory is partly supported by the fact that the over expression of MexAB-OprM and MexXY-OprM efflux pumps has already been linked to resistance in *P. aeruginosa* in multiple antimicrobial agents such as aminoglycosides (Hocquet et al., 2003). One interesting study done by Bahador et al. (2018) found that resistance to colistin in *A. baumannii* isolates is linked to the increase in virulence factors such as biofilm formation in burn patients. This renders the treatment of such MDR more challenging, as both resistance to colistin and virulence factors must be tackled at once (Bahador et al., 2018). Furthermore, two studies reported the isolation of colistin resistant *A. baumannii* and *K. pneumoniae* with mutations in the pmrB and mgrB genes, respectively (Haeili et al., 2017, 2018) (Table 3).

**TABLE 3:** Mechanisms of colistin resistance described in GNB in the Middle East.

**Country**	**Origin**	**Species**	**Sequence type**	**Mechanism of colistin resistance**	**References**
Iran	Clinical samples	*A. baumannii*		pmrB^∗^	Haeili et al., 2018
		*A. baumannii*		pmrA/B^∗^	Sepahvand et al., 2016
		*K. pneumoniae*		mgrB^∗^	Haeili et al., 2017
		*P. aeruginosa*		MexAB-OprM/MexXY-OprM^∗∗^	Goli et al., 2016
Lebanon	Clinical samples	*K. pneumoniae*	ST268	mgrB^∗^	Okdah et al., 2017
		*K. pneumoniae*	ST2296	mgrB^∗^, PhoQ^∗^	
		*K. pneumoniae*	ST348	pmrA/B^∗^	
	Poultry	*E. coli*	ST515	*mcr-1*	Dandachi et al., 2018c
	Swine	*E. coli*		*mcr-1*	Dandachi et al., 2018b
Palestine	Clinical samples	*K. pneumoniae*		mgrB^∗^	Olaitan et al., 2014a
		*K. pneumoniae*	ST512, ST76	mgrB^∗^	Lalaoui et al., 2019
Bahrain	Clinical samples	*E. coli*	ST648, ST224	*mcr-1* on IncI2	Sonnevend et al., 2016
Qatar	Clinical samples	*E. coli*	ST95	*mcr-1* on IncHI2	Forde et al., 2018
United Arab Emirates	Clinical samples	*K. pneumoniae*	ST147	mgrB^∗^	Sonnevend et al., 2017a
		*E. coli*	ST131	*mcr-1* on IncI2	Sonnevend et al., 2016
Oman	Clinical samples	*E. coli*	ST10	*mcr-1* on IncI2	Mohsin et al., 2018
KSA	Clinical samples	*K. pneumoniae*	ST974, ST37, ST709, ST348, ST37	PhoP^∗^	Uz Zaman et al., 2018
		*K. pneumoniae*	ST14, ST15, ST16, ST22, ST48, ST101, ST152, ST307	mgrB^∗^	
		*K. pneumoniae*	ST15	mgrB^∗^, phoP^∗^	
		*E. coli*	ST68	*mcr-1* on IncHI2	Sonnevend et al., 2016
Egypt	Clinical samples	*A. baumannii*		pmrCAB^∗^	Abdulzahra et al., 2018
	Animal	*E. coli*	ST10	*mcr-1*	Khalifa et al., 2016
		*E. coli* O157		*mcr-1*	Lima Barbieri et al., 2017
		*E. coli* O158		*mcr-1*	

*^∗^Mutations. ^∗∗^Efflux pump over-expression.*

### Carriage of ESBL/Carbapenemase Producers

The main concern of MDROs intestinal carriage is the acquisition of MDRO caused infections with limited therapeutic options (Magwenzi et al., 2017). In addition, as the carriage can last from months to years, the asymptomatic colonization of MDROs constitute a potent reservoir for transmission and dissemination (Decker et al., 2018).

In Iran, one study reported an 18.3% rectal carriage rate of ESBL *K. pneumoniae* among ICU patients and outpatients. The main mechanism of resistance was the production of CTX-M-15 detected in 86.3% of isolated strains (Aghamohammad et al., 2018). MLST analysis revealed that isolates of *K. pneumoniae* belonged to 16 different STs with a predominance of ST15, ST147, and ST16 (Aghamohammad et al., 2018). ST15 *K. pneumoniae* is widely associated worldwide with the production of CTX-M-15 (Lee et al., 2011; Rodrigues et al., 2014; Caneiras et al., 2019). On the other hand, carbapenem resistant Enterobacteriaceae (CRE) colonization in Iranian inpatients was associated with 3rd generation cephalosporins, meropenem, colistin, and vancomycin exposure. This is in addition to ICU hospitalization, urinary catheter, mechanical ventilation, recent surgery, patient transfers from another hospital/unit and being male (Solgi et al., 2017a). Isolated CRE included, NDM and OXA-48 producing *K. pneumoniae*, *E. coli*, *E. cloacae*, and *P. mirabilis* (Solgi et al., 2017a).

In Turkey, the rectal carriage of CRE (OXA-48, NDM-1, and IMP *K. pneumoniae*) as well as carbapenem resistant non-fermenters (CR-NF) was reported in several studies (Alp et al., 2013; Karaaslan et al., 2016). In one study, only carbapenem intake was associated with OXA-48/IMP producing *K. pneumoniae* infections (Zarakolu et al., 2016). An interesting clinical experience was the one reported by Poirel et al. (2014) when an outbreak of carbapenem resistance was suspected with the first isolation of two similar carbapenem resistant *E. cloacae* from two patients’ residing in the neonatal ICU (NICU). Accordingly, nasal and rectal screening was performed for all NICU patients. In addition, contact isolation precautions were implemented as well as an intensive infection control program was performed for all staff personnel. Subsequently, after 1 month, no infection/colonization with CRE was observed (Poirel et al., 2014). This emphasizes the significance of the microbiology laboratory and infection control unit’s cooperation in preventing the dissemination of CRE (Poirel et al., 2014). A more recent study, also conducted in a Turkish NICU, showed that ages less than 1 year, carbapenem administration, presence of underlying diseases, urinary catheterization, and nasogastric tube placement were independent risk factors for CRE colonization. In this study, CRE included OXA-48, IMP, NDM *K. pneumoniae*, *E. coli*, and *E. cloacae* (Karaaslan et al., 2016). On the other hand, CR-NF carriage (NDM, IMP-1, OXA-23, OXA-24, and OXA-58 producing *A. baumannii*) was correlated to an ICU stay, ampicillin carbapenem use, mean daily antibiotic use, presence of underlying diseases, surgical intervention and nasogastric tube placement (Karaaslan et al., 2016).

In Lebanon, two studies addressed the rectal carriage of ESBL producing Enterobacteriaceae in nursing home residents in Beirut and Tripoli in the north (Jallad et al., 2015). In Beirut, constipation and antibiotic intake were independent risk factors for ESBL carriage (Jallad et al., 2015); whereas in Tripoli, only antibiotic administration was found (Dandachi et al., 2016). Nursing homes are community facilities where MDROs can easily emerge and spread due to the uncontrolled or unprofessional prescription of antibiotics and inadequate environmental decontamination, waste disposal, and hygiene practices (Dandachi et al., 2016). Another study conducted in healthy infants showed that CTX-M-15, CTX-M-9, and CTX-M-2 positive Enterobacteriaceae are prevalent in the Lebanese community (Hijazi et al., 2016). Hospital birth, cesarean delivery, being formula-fed and being male are important risk factors for ESBL colonization in this category. In this report, proper hygiene was associated with a colonization rate decrease (Hijazi et al., 2016). On the other hand, Christophy et al. (2017) reported a high prevalence of carbapenem resistance fecal carriage in cancer patients undergoing chemotherapy. The carbapenem resistant strains included mainly OXA-48/CTX-M *E. coli*, OXA-48 *E. cloacae* and VIM *Pseudomonas stutzeri*.

In Israel, a study conducted at a rehabilitation center revealed the patient’s rectal carriage of CTX-M-27, CTX-M-15, CTX-M-14, CTX-M-39, CTX-M-55, SHV-5, SHV-12, and CMY-4 and CMY-2 producing *E. coli* strains of diverse sequence types including ST131 (Izdebski et al., 2013). In parallel, among patients admitted to a teaching hospital in one study, 8% were carriers of ESBL. The risk factors for this colonization were female sex and recent antibiotic intake. On the other hand, 21% of admitted patients acquired ESBL carriage. The latter was significantly associated with being older than 65 years and having an extended spectrum beta lactam antibiotic intake (Friedmann et al., 2009). Additionally, in clinical settings, one study raised concern about the real efficiency of antibiotic prophylaxis post-bowel surgery on ESBL carriage and subsequent infection. This is because in this report, immunosuppressive therapy and antibiotic use in the previous 3 months were independent risk factors for ESBL rectal carriage in this patients’ category (Pfeffer et al., 2016). Moreover, in this country, the carriage of carbapenem resistance was significantly higher to ESBL positive Enterobacteriaceae in view of the number of reports. In one study, the carriage of CR-KP was significantly associated with a prolonged hospital stay, room sharing with a previously known carrier and residency in a high carrier ward (Ben-David et al., 2011). In fact, in their study, Wiener-Well et al. (2010), found that during the surveillance of CR-KP carriage in hospitalized patients, isolated strains had identical PFGE patterns showing a clonal origin. The authors argued that strict isolation of carriers might help reduce the transmission of the CR-GNB from one patient to another (Wiener-Well et al., 2010). In another study, the CR-KP carriage was dependent on recent surgery and a sequential organ failure assessment (SOFA) score (Debby et al., 2012). Other described risk factors for CR-KP intestinal carriage include diaper use, length of hospital stay and vancomycin use (Wiener-Well et al., 2010). Adler et al. (2015) reported an increase in the rectal carriage of KPC producing *K. pneumoniae* (represented by ST258) in a post-acute care hospital (PACH) from 65% in 2008 to 80% in 2013. The acquisition source of more than 50% of the carriers was the PACH itself (Adler et al., 2015). Moreover, one report showed that the duration of CRE carriage can last for 3 months, 6 months and even up to 1 year. The carriage duration was affected mainly by repeated hospitalization and the isolation of a clinical and not surveillance positive culture (Zimmerman et al., 2013). One explanation for this finding is that recurrent hospitalization often represents re-infection and flags more severely ill people who need more time to eradicate CRE (Zimmerman et al., 2013). Furthermore, CRE infected patients might have larger loads of CRE compared to those who are only colonized with; contributing subsequently to longer periods of continuous carriage (Zimmerman et al., 2013). Interestingly, one study assessed the risk factors responsible for the development of CRE infection after CRE colonization. These latter included ICU admission, antibiotic intake (especially fluoroquinolones and metronidazole), diabetes mellitus and central venous catheter insertion (Schechner et al., 2013). The identification of these factors are important in order to predict CRE infections and direct accordingly antibiotic empirical therapy (Schechner et al., 2013). Other carbapenem resistant species detected in Palestinian colonizers include VIM (VIM-1, VIM-35) producing *Aeromonas* species and NDM-1/OXA-10 positive *P. rettgeri* (Adler et al., 2014; Olaitan et al., 2016b). In Jordan, one study reported the rectal carriage of CTX-M-15, CTX-M-2, and CTX-M-1 *E. coli* in infants less than 1 year of age to the Pediatric unit in a hospital in Amman (Badran et al., 2016).

In the Gulf region, Dashti et al. (2010b) reported the detection of a single ESBL producing *E. coli* clone in blood cultures of neonates and health care workers’ (HCW) hand in a Kuwaiti hospital. This highlight the important role that the health care personnel can play as vectors and reservoirs from which bacterial resistance can spread. This is especially true when non-adherence to proper sanitation and hand hygiene occur. In Qatar, only one study reported the fecal carriage of MDR ESBL *E. coli* in food handlers (Eltai et al., 2018c). This finding is of public health concern, since MDROs can be silently transmitted to the general community via contaminated food, contributing thus further to the dissemination of bacterial resistance (Eltai et al., 2018c).

In KSA, only two studies reported the rectal carriage of MDROs in the clinical settings. These latter included ICU patients carrying of highly diverse OXA-23 *A. baumannii*, CTX-M-15 *K. pneumoniae*, and NDM, VIM producing *P. aeruginosa* (Aljindan et al., 2015; Abdalhamid et al., 2016). Indeed, the rectal colonization of MDROs was mostly addressed in the Hajj period. One study reported in 2013 a significant CTX-M intestinal carriage in pilgrims with the rate of the latter increasing from 10.08% before Hajj to 32.56% post Hajj (Leangapichart et al., 2016a). In the same context, the same author reported similar findings in 2014. The acquisition rate of ESBL producers did not significantly differed between the 2 years (Leangapichart et al., 2017). Indeed, Leangapichart et al. also found that there was a difference not only at the level of intestinal colonization rate but also at the level of the bacterial diversity detected. For instance, *A. baumannii* strains were isolated from 26 rectal specimens and 16 pharyngeal one’s post Hajj while none was detected in the samples collected prior to Hajj travel (Leangapichart et al., 2016c). It is worth mentioning the detection of one *A. baumannii* strain that is carbapenem resistant and produced the OXA-72 carbapenemase post Hajj. Likewise, an *E. coli* positive for blaNDM-5, blaCTX-M-15 was also detected after Hajj travel (Leangapichart et al., 2016c). These data emphasize the role of this season as a mediator of bacterial resistance dissemination in the KSA and worldwide. More effort is warranted for the improvement of the public health conditions during this period of the year. Moreover, recent Hajj travel should be taken into consideration when pilgrim patients are admitted to hospitals in their hometown in order to control for the introduction of new MDROs to clinical settings. However, more studies are needed in order to characterize “recent Hajj travel” as a risk factor for MDROs fecal carriage.

In Egypt, fecal carriage of ESBL producing GNB was detected in hospitalized patients. These included CTX-M (CTX-M-15, CTX-M-14, CTX-M-2, and CTX-M-grp9) SHV and TEM ESBL type (Khalaf et al., 2009; Fam et al., 2015). Fouda et al. (2016) found that ESBL carriage was associated with increased mortality in ICU admitted patients. In the same context, two studies reported the intestinal carriage of ESBL and AmpC beta lactamases in HCW in two hospitals (Abdel Rahman et al., 2010; Bassyouni et al., 2015). As already mentioned, HCW constitute a potent reservoir of bacterial resistance when infection control measures and proper hand hygiene are lacking in a clinical center (Bassyouni et al., 2015). Furthermore, NDM-1 positive ST267 *A. baumannii* were isolated from hospitalized patients during a rectal screening surveillance in this same country (Krahn et al., 2016).

## Distribution of Multi-Drug Resistant Organisms in Animals

### ESBL/AmpC Producers

In the Middle East, studies from Egypt reported the detection of TEM, SHV, CTX-M-9, CTX-M-15, and OXA-7 producing *E. coli* strains in broiler farms. The plasmid mediated AmpC beta lactamase genes blaCMY-2 and blaDHA-1 were also observed (Moawad et al., 2018). Furthermore, studies done on poultry hatcheries revealed similar results where blaTEM, followed by blaSHV, blaMOX-like, blaCIT-like, and blaFOX were the most common beta lactamase genes detected (Osman et al., 2018). BlaCTX-M-15 has also been reported in Egyptian poultry with other β-lactamase-encoding genes such as blaTEM-104, blaCMY-2, and blaOXA-30 in *E. coli* strains including the sequence type ST131 (Ahmed et al., 2013; Abdallah et al., 2015a; Ramadan H.H. et al., 2018). Multidrug-resistant *E. coli* O25b:H4 ST131 has been reported to be spread worldwide in humans, companion animals and livestock (Ahmed et al., 2013). Another study on chickens in Egypt, reported other ESBL types including blaTEM-57, blaSHV-12, blaCTX-M-14 (El-Shazly et al., 2017). As for pathogenic bacteria, two studies reported the detection of TEM ESBL type in *Salmonella* species isolated from chicken meat as well as from pigeons (Ahmed H.A. et al., 2016; Abdeen et al., 2018). Indeed, one of the main contributors to this high prevalence of ESBL/AmpC producers in the Egyptian poultry sector is the misuse of antibiotics. According to El-Shazly et al. (2017), many farmers in Egypt tend to use cefotaxime injections (a 3rd generation cephalosporin banned in poultry) to treat diseases in chicken (such as colibacillosis) after the failure of other antimicrobial treatments like fluoroquinolone and aminoglycosides. In addition, due to the low cost of antibiotics, many veterinarians still over-use antibiotics such as tetracycline, quinolone and beta lactams to treat and prevent zoonotic diseases and growth promotion (Braun et al., 2016; Hakim et al., 2017). Moreover, in pets, blaTEM along with blaSHV, blaPSE-1 and blaCTX-M were detected in *E. coli* strains isolated from dogs in in the same country (Aly et al., 2012). In cattle, TEM, SHV (SHV-11, SHV-27), and CTX-M-15 were detected in *E. coli* and *K. pneumoniae* strains (Hammad and Shimamoto, 2011; Braun et al., 2016). Another report on dairy calves, reported the detection of blaCMY-2 and blaSHV-12 genes in *Salmonella* spp. including *S. enterica serovars enteritidis* and *S. typhimurium* (Ahmed et al., 2009). Other studies in the Egyptian dairy products revealed the presence of CTX-M-variants (CTX-M-15, CTX-M-104, CTX-M-3), TEM-52, SHV-12, and CMY-2 producing *E. coli* strains (Ahmed and Shimamoto, 2015; Ombarak et al., 2018). Other ESBL (including OXA-10 and SHV-28) and AmpC producing GNB detected in the Egyptian bovine sector include *Klebsiella oxytoca* and *C. freundii* (Ahmed and Shimamoto, 2011).

In Palestine, blaCTX-M (including CTX-M-1, CTX-M-9) and SHV-12 were the only ESBL types detected in *E. coli* strains isolated in Chicken (Qabajah et al., 2014). Similarly, these ESBL types were detected in cattle in Israel (Adler et al., 2016). In Lebanon, a recent nationwide study conducted in chicken farms, found a considerable number of ESBL and AmpC producing GNB. These included mainly blaCTX-M, blaTEM and blaCMY genes (Dandachi et al., 2018). On the other hand, Diab et al. (2016) reported the dissemination of CTX-M-15 producing *E. coli* in Lebanese cattle. One more recent study conducted by Dandachi et al. (2018b) found that CTX-M followed by CMY are the most common beta lactamases detected in *E. coli* strains isolated from Swine farms. Both in cattle and poultry, MLST analysis revealed high variety of sequence types in isolated *E. coli* strains with some of them previously described in the literature as being common to animals as well as to humans (ST10, ST617, ST58, ST69, ST155, and ST156) (Diab et al., 2016; Dandachi et al., 2018). This emphasizes the role of livestock in the dissemination of MDROs in the one health concept.

In Turkey, CTX-M-15 was detected in *E. coli* strains belonging to the B1 phylogenetic group isolated from cattle with bovine mastitis (Pehlivanoglu et al., 2016). Furthermore, one study targeting MDROs in dogs reported the detection of CTX-M-15, BlaCMY-2, blaCTX-M-3, blaCTX-M-1, and blaSHV-12 in *E. coli* isolates with A1 and D2 being the most common phylogenetic groups identified. In this report, ST131/B2 *E. coli* positive for CTX-M-15 were detected. This clone is a human pandemic one that can possibly be transmitted to humans via direct or indirect contact with companion animals (Aslantas and Yilmaz, 2017).

In the gulf and specifically in the KSA, blaSHV and blaTEM were reported in *E. coli* strains isolated from poultry (Altalhi et al., 2010; Abo-Amer et al., 2018). Furthermore, ESBL and AmpC producers were detected in the Qatari chickens and green turtles in Oman, respectively (Al-Bahry et al., 2012; Eltai et al., 2018a). In Iran, *E. coli* strains producing blaSHV were isolated from raw milk and dairy products across the country (Ranjbar et al., 2018). Furthermore, this same gene was detected in Uropathogenic *E. coli* strains isolated from dogs (Yousefi and Torkan, 2017).

### Carbapenem and Colistin Resistance

**FIGURE 2:**
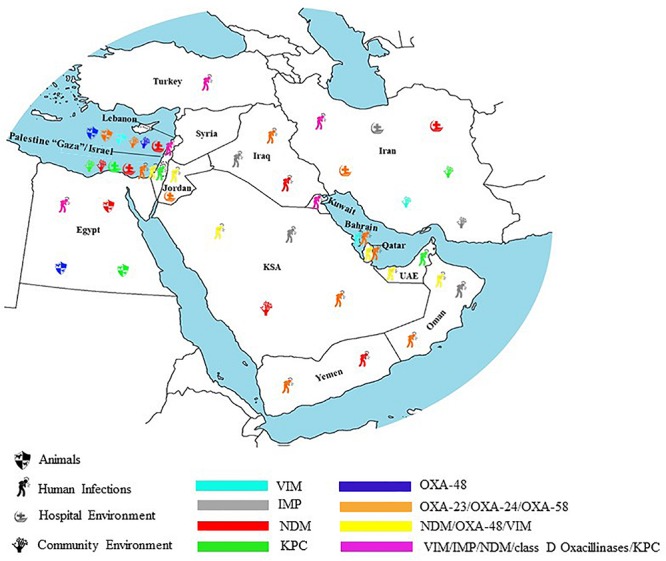
Geographical distribution of carbapenemases in humans, animals, hospital, and community environment in the Middle East.

Unlike ESBL and AmpC producers, carbapenemase producing GNB are not widely spread in animals of the Middle East (Figure 2). Al Bayssari et al. (2015b) reported the isolation of ST38 *E. coli* positive for the blaOXA-48 from fowl in Lebanon. Furthermore, they detected VIM-2 carbapenemase in *P. aeruginosa* and blaOXA-23/blaOXA-58 genes *A. baumannii* strains isolated from cattle, swine and fowl (Al Bayssari et al., 2015a). Furthermore, Rafei et al. (2015a) reported the isolation of OXA-143 *A. baumannii* and OXA-24 *A. pittii* from a horse and a rabbit oral cavity, respectively. In Egypt, carbapenem resistant *K. pneumoniae* (CR-KP) have been isolated from broilers, drinking water and workers in chicken farms (Abdallah et al., 2015a). The genes responsible for resistance were blaKPC, blaOXA-48, and blaNDM. In cattle, OXA-48 and OXA-181 producing *E. coli* were detected (Braun et al., 2016).

**FIGURE 3:**
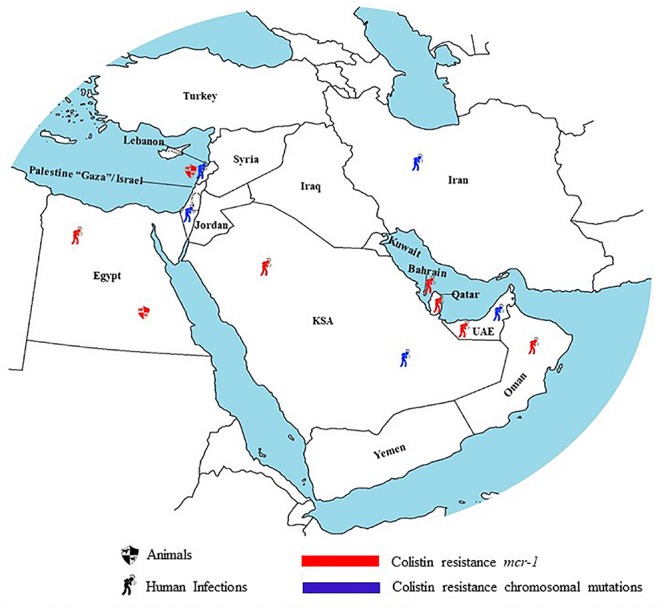
Geographical distribution of colistin resistance in humans and animals in the Middle East.

As for colistin resistance (Figure 3), in Egypt, it is known that colistin is used in animal husbandry in farms, calves, poultry, and rabbits (Lima Barbieri et al., 2017). In poultry for example, colistin has been used for colibacillosis. Colistin resistant avian isolates of *E. coli* that have been found in Egyptian farms imply that the overuse of colistin in the farming industry can indeed have participated in the emergence of colistin resistance in Egypt (Lima Barbieri et al., 2017). Indeed, in samples collected from both poultry and cattle, the *mcr*-1 gene was detected (Khalifa et al., 2016; Lima Barbieri et al., 2017). In cattle, *mcr-1* was harbored by an ST10 *E. coli* strain (Khalifa et al., 2016). In the light of the One Health concept, those resistant strains can potentially enter the human food chain and result in treatment challenging infections that pose a serious threat to the Egyptian population. This is especially relevant in a country like Egypt which is known to struggle with infectious diseases and poor control of antibiotic use (Khalifa et al., 2016). On the other hand, Lebanon is considered one of the more recent countries in which colistin resistance has emerged. Dandachi et al. (2018c) reported the first detection of ESBL/*mcr*-1 ST515 *E. coli* strain isolated from chicken in the South of Lebanon. *mcr*-1 *E. coli* strains were also detected in Lebanese pigs (Dandachi et al., 2018b).

## Distribution of Multi-Drug Resistant Organisms in the Environment

### Community Environment

In Iran, a study addressing MDROs in surface water resources found a high prevalence of CTX-M, TEM, SHV, and OXA genes among isolated *E. coli* strains (Ranjbar and Sami, 2017). In another study conducted in community water filtering systems, *P. aeruginosa* producing *bla*VIM-1, *bla*NDM, and *bla*IMP-1 were detected (Mombini et al., 2019). In Turkey, ESBL producing *E. coli* strains were detected in samples collected from Orontes River. The most common ESBL type was CTX-M-15 with the majority co-harboring the sulfonamide resistance gene *sul* (Kurekci et al., 2017).

In Lebanon, Diab et al. (2018) targeted the issue of MDROs contamination in three water sources: estuaries, wells and spring water (Diab et al., 2018). It was found that in estuaries, 17 ESBL and four carbapenemase producing GNB were isolated. The most prevalent ESBL gene was the bla-CTX-M-15 followed by CTX-M-55, CTX-M-14, and SHV-12. IncF type plasmid was the most common plasmid type detected among ESBL. In parallel, carbapenem resistance was mediated by the presence of bla-OXA-48 carried by the IncL plasmid and the bla-OXA-244 carried by the IncHl2 plasmid. On the other hand, only ESBL producers were detected in wells and spring water. These included the blaCTX-M-15 gene located on an IncF plasmid (Diab et al., 2018). In another study, ESBL producing *E. coli* were addressed in a refugee camp and from river effluents (Tokajian et al., 2018). The prevalence of ESBL was similar in both groups (53.11% versus 49.1%). However, the latter presented with different phylogroups and sequence types; in addition, refugee camp isolated strains had higher resistance rates toward aminoglycosides, fluoroquinolones and trimethoprim-sulfamethoxazole (Tokajian et al., 2018). In this study, the most common ESBL types detected in both sources were CTX-M-15, CTX-M-27, CTX-M-14, and CTX-M-9 (Tokajian et al., 2018). Furthermore, it is worth noting the detection of OXA-72 producing *Acinetobacter calcoaceticus* in vegetables in Lebanon. The presence of MDROs in vegetables could be the result of direct animal contamination or indirect environmental contamination with soil or irrigation water (Al Atrouni et al., 2015, 2016b). Altogether, due to the lack of proper water treatment systems in Lebanon, water sources have become major environmental reservoirs for MDROs. In Israel, one study reported the detection of *bla*KPC and *bla*NDM-1 producing Enterobacteriaceae (mainly *K. pneumoniae* and *E. cloacae*) in sewage systems (Meir-Gruber et al., 2016).

In the KSA, *bla*NDM-1 ST101 *E. coli* was isolated from wastewater (Mantilla-Calderon et al., 2016). In addition, Alghoribi et al. (2015) reported the detection of CTX-M producing *E. cloacae* from a community sewage.

### Hospital Environment

In Iran, TEM and SHV producing *P. aeruginosa* were detected in hospital environmental samples (Gholami et al., 2017). Another study, in which the samples were taken from an ICU setting, revealed that among the *Klebsiella* species isolated, the majority were carbapenem resistant *K. pneumoniae*. The ICU’s contaminated areas are a great source for the spread of MDROs in their surroundings (Moghadampour et al., 2018b). In isolated *K. pneumoniae* strains, the highest resistance rate was observed for β-lactam antibiotics and the lowest resistance rate was toward tigecycline. In this study, blaOXA-48 was the most prevalent carbapenemase detected, followed by blaNDM (Moghadampour et al., 2018b). Furthermore, various hospitals have been suffering from antibiotic resistant *A. baumannii*. In one study, several isolates where taken from different parts of the hospital with the greatest number of *A. baumannii* coming from the ICU. These strains carried the OXA-23 and OXA-24 genes (Shamsizadeh et al., 2017). The prevalence of CRKP has been another problem in Iran, especially on hospital equipment where in one study 34 CRKP were isolated (Moghadampour et al., 2018b). Aliramezani et al. (2016) also reported the detection of carbapenem resistant GNB (OXA-23 *A. baumannii*) in instruments that are frequently used for the care of patients such as dressing sets, suction tubes, hand-washing sinks and faucets. The occurrence of MDROs in the aforementioned surfaces raise the chance of acquisition and HGT of resistance genes among patients, health care personnel and visitors, creating thus a significant source of hospital acquired infections (Aliramezani et al., 2016). Moreover, other studies in Iran reported the isolation of carbapenemase producing *A. baumannii* (OXA-23, OXA-24, and OXA-58) from hospital environmental samples (Kulah et al., 2010; Salehi et al., 2018). Of special interest is the detection of OXA-23 and CTX-M-32 genes in air samples collected from operating theaters, ICUs, surgery and internal medicine wards (Mirhoseini et al., 2016). This study unveiled another source of the environmental route of MDROs transmission. Not only is area disinfection warranted but the application of efficient working ventilation and air quality monitoring systems is also highly needed (Mirhoseini et al., 2016).

In Turkey, only one study conducted in hospitals reported the detection of ESBL *K. pneumoniae* in broiler distilled water in a NICU (Hosbul et al., 2012). In Israel, a study conducted inside a general hospital with emphasis on the bacteria present on wheelchairs, found that *P. aeruginosa* followed by *A. baumannii* were the most common MDROs detected and exhibited resistance to all antibiotics tested, especially in the samples taken from wheelchairs in the surgery department (Peretz et al., 2014a). Interestingly, one study assessed the prevalence of environmental contamination of CRE in the vicinity of 34 carriers. Among these 26 were spreaders with a group of six being responsible for 79% of the environmental CRE detected. Statistical analysis revealed that fecal continence was the sole independent factor associated with CRE non-spread. On the other hand, high rectal colonization with these MDROs in addition to being admitted with a respiratory disease were the only independent risk factors for CRE shedding (Lerner et al., 2015). Therefore, imminent protocols must be set to minimize contamination and spread of infections in all hospital settings.

In Lebanon, the most discussed source of MDROs contamination are of a human source whether directly or indirectly through hospital sewage. In addition to that, water is considered a pivotal driver of contamination because it acts as a reservoir for MDROs and receives them from multiple sources. Furthermore, Daoud et al. (2018) found that isolates of *E. coli* from the hospital wastewater produced CTX-M ESBL at a rate of 81.5% in one hospital and 94.4% in another. SHV beta-lactamases were produced by 55.6 and 44.4% of the isolates in each hospital, respectively. In the same context, Daoud et al. (2017), addressed the MDROs in two hospital sewage treatment plants. In this latter, ESBL and AmpC producers including *E. coli*, *E. cloacae*, *Klebsiella* spp., and *Serratia odorifera* were isolated. The most common ESBL types found were CTX-M followed by SHV, TEM, and OXA. Furthermore, only one CRE was detected (Daoud et al., 2017).

In Iraq, only one study revealed the detection of ESBL *Klebsiella* spp. and *E. coli* producing CTX-M-15, AmpC beta lactamases and SHV ESBL types, respectively, in hospital environmental samples (Huang et al., 2012). Interestingly, Obeidat et al. (2014) reported the isolation of carbapenem resistant *A. baumannii* (OXA-23 and OXA-24 producing) from a hospital environment as well as from patients respiratory tracts; the high similarity of MDR patterns suggest the persistence of these MDRO in the environment is responsible for their high colonization rates detected in the respiratory tracts of ICU patients.

In the gulf, one study in the KSA found ESBL producing GNB in hospital sewage. The sewage tank might play a significant role in the dissemination of MDROs, especially if it enters the sea and beach recreational activity areas, subsequently affecting the community population (Alghoribi et al., 2015).

## Antibiotic Use in the Middle East

### Clinical Setting and Community

Nowadays, antibiotics are among the most common drugs prescribed worldwide. Between 2000 and 2010, antibiotic consumption increased by 20 billion standard units (Auta et al., 2019). The growing use of antibiotics through prescriptions or non-prescriptions is linked to the spread of MDROs, therefore causing a global public health concern (Auta et al., 2019).

In the Middle East, studies have shown that SM is highly prevalent. In Iran, SM ranges from 35.4 to 83%, 32 to 42% in Lebanon, 32 to 62% in Jordan, 98% in Palestine, 85% in Syria. In the Gulf, SM rates were as high as 89.2% in the United Arab Emirates, 48% in the KSA and 60% in Yemen (Khalifeh et al., 2017). This is unlike European countries where “over-the-counter” access to antibiotics is strictly regulated, thus resulting in SM prevalence rates ranging from 1 to 4% only (Alhomoud et al., 2017). The major types of antibiotics sold over-the-counter in the Middle East include penicillin, macrolides, cephalosporins, fluoroquinolones, and tetracycline (Alhomoud et al., 2017). The main reason behind the common practice of SM in Middle Eastern countries is the lack of strict policies controlling the sale of antibiotics without a prescription from pharmacies. This is in addition to the low economic status and lack of health care insurance that push individuals to retrieve medications from pharmacists to avoid consultations costs (Alhomoud et al., 2017).

Besides SM, inappropriate use of antibiotics in hospitals is another reason behind the dissemination of MDROs in the Middle East. Indeed, ASP, although implemented in some hospitals in several countries such as Lebanon, Jordan, Palestine, the KSA, the United Arab Emirates, Bahrain, Qatar, and Oman; these are still in their infancy (Nasr et al., 2017). Barriers for the implementation of ASP in the Middle East are divided mainly into two levels: individual and hospital barriers. Individually speaking, physicians often lack up-to-date knowledge for appropriate antibiotic use and resistance, reluctance for antibiotic prescription other than the usual and fear of patient complications especially in very sick patients are other individual barriers (Alghamdi et al., 2018). On the other hand, lack of expertise, unavailability of some antibiotics, lack of education/training for appropriate usage of antibiotics and antimicrobial resistance as well as a lack of financial, administrative and management support are all barriers against the implementation of ASP at the hospital level (Alghamdi et al., 2018).

Further research assessing the knowledge, attitude and practices of antibiotic prescription among expatriates is crucial for the adoption of successful programs, in order to promote the rational use of antimicrobial agents in the Middle East (Alhomoud et al., 2017). Furthermore, hospital leadership is paramount to ensure policies’ enforcement, in collaboration with physicians and other stakeholders (Alghamdi et al., 2018). On the other hand, as for SM, enforcing regulatory measures that restrict antibiotic access to “prescribed-only,” developing national resistance as well as antibiotic consumption surveillance systems can all help in reducing the rates of SM (Alhomoud et al., 2017). This is definitely in addition to public awareness campaigns addressing the proper use of antibiotics in addition to the dangers of their inappropriate use and over-intake (Alhomoud et al., 2017).

### Animals and Environment

Unfortunately, it is evident that MDROs are nowadays disseminated in animals and the environment as it has been reported worldwide (Rizzo et al., 2013; Alonso et al., 2017; Dandachi et al., 2018a). Similar to humans, among other factors, the un-regulated use of antibiotics in veterinary medicine is the main cause for MDRO dissemination (Guerra et al., 2014). Besides treatment, in animals, antibiotics are also given as growth promoters and for prophylaxis. As growth promoters, this practice is no longer applied in the European Union, but it persists in North America and other countries (Economou and Gousia, 2015). MDROs in animals can be transmitted to humans via direct/indirect contact or via the surrounding environment (Pomba et al., 2017). Despite their importance in the transmission chain, surveillance studies addressing MDROs in these two ecosystems in the Middle East are scarce. As shown in Figures 1–3, epidemiological studies describing the dissemination of MDR in animals and the environment were conducted in only six out of the 15 countries. The level of antibiotic consumption in livestock is unknown and thus policies to control the misuse and overuse of antimicrobial agents in veterinary medicine are not yet in place. Furthermore, the role of the environment in the transmission route is also unknown in this region of the world. In the environment, resistant bacteria can spread either due to the shedding of MDROs from human/animal waste or via the antibiotic selective pressure created by antimicrobial release in livestock and human waste streams (Dar et al., 2016). In the one health concept “the health of people is connected to the health of animals and the environment” (Centers for Disease Control and Prevention [CDC], 2018). Researchers in Middle Eastern countries are therefore recruited to conduct studies to fill the gaps of epidemiological distribution of MDROs as well as antibiotic consumption in ecosystems other than humans. Furthermore, the implementation of an integrated human-animal surveillance system where samples are obtained from both humans, livestock and the environment and then processed with a synchronized monitoring system can assist these speculations (Manyi-Loh et al., 2018). The first worldwide system integrating humans and animals was the “DANMAP” (Danish Integrated Antimicrobial Monitoring and Resistance Program) which addresses the problem of MDR in livestock, food of animal origin and people (Dar et al., 2016).

## Conclusion

This review shows the extensive dissemination of ESBL and carbapenemase producing GNB in Middle Eastern hospitals. The prevalence of these MDROs is less well documented in animals and the environment. However, studies reported that ESBL is common in livestock whereas carbapenemases are scarce. In the environment, to some extent both groups (ESBL and carbapenemases) were reported equally. This emphasizes that the environment plays a double route in the transmission of resistant organisms from humans to animals and vice versa. In some countries especially in the gulf, nothing is known about the spread of MDROs in animals nor the environment; therefore, a clear conclusion cannot be drawn. One major mediator of MDROs spread in the Middle East is the recent population mobilization due to the socio-economic crisis and the Syrian war. This conflict promotes the introduction of resistance genes not previously reported in those countries. The emergence of colistin resistance is another major issue. In most of the epidemiological studies, colistin susceptibility is assessed by the Kirby-Bauer technique. This method is unreliable and might underestimate the real prevalence of colistin resistance in all ecological niches.

## Author Contributions

ID, AC, JH, and JM wrote the manuscript. ZD corrected the manuscript. All authors approved and revised the final version of the manuscript.

## Conflict of Interest Statement

The authors declare that the research was conducted in the absence of any commercial or financial relationships that could be construed as a potential conflict of interest.
